# Natriuretic Peptide Receptor-C (NPR3) in Pulmonary Hypertension Associated with Chronic Lung Disease: A PRISMA-ScR Scoping Review of Diagnostic, Prognostic, Mechanistic, and Therapeutic Evidence

**DOI:** 10.3390/jcm15197429

**Published:** 2026-09-24

**Authors:** Emmanuel Eroume A Egom, Adamou Mfopa, Njimona Ibrahim, Mountchissi Célestin, Medou Mba Fabrice, Lum Abenwi Ambe, René Kamgang

**Affiliations:** 1 Laboratory of Human Metabolism and Non-Communicable Diseases, Institute of Medical Research and Medicinal Plants Studies (IMPM), Yaoundé P.O. Box 13033, Cameroon; mfopadam2000@gmail.com (A.M.); njimona@impm-cm.org (N.I.); celestinmountchissi87@gmail.com (M.C.); fabricemmf@yahoo.com (M.M.F.); lumabienwiambe@yahoo.com (L.A.A.); gemskruy@yahoo.fr (R.K.); 2 Institut du Savoir Montfort (ISM), Ottawa, ON K1K 0T2, Canada; 3 Harmony Health Physical and Spiritual Wellness Center/Harmony Care, Hartford, CT 06105, USA

**Keywords:** pulmonary hypertension, chronic lung disease, natriuretic peptide receptor-C, NPR3, C-type natriuretic peptide

## Abstract

**Background/Objectives:** Pulmonary hypertension associated with chronic lung disease remains difficult to diagnose, phenotype, and manage because vascular remodeling, parenchymal lung injury, hypoxemia, inflammation, fibrosis, and right ventricular dysfunction often coexist. Natriuretic peptides are widely used as cardiopulmonary biomarkers, but the receptor-level role of natriuretic peptide receptor-C, encoded by NPR3, remains incompletely defined. This scoping review mapped original empirical evidence on NPR-C/NPR3 and related C-type natriuretic peptide biology in pulmonary hypertension, chronic lung disease, pulmonary vascular remodeling, and right ventricular adaptation. **Methods:** This scoping review was conducted and reported in accordance with PRISMA-ScR, with applicable PRISMA 2020 reporting elements addressed where relevant to a scoping review. Searches of PubMed/MEDLINE and APA PsycINFO identified 72 records. After removal of 2 duplicates, 70 records underwent title and abstract screening. Forty-two records were excluded at this stage, and 28 reports were sought for full-text retrieval. All reports were retrieved and assessed for eligibility. Eleven reports were excluded at full-text review, leaving 17 original studies for inclusion in the evidence map. Reviews, meta-analyses, editorials, commentaries, protocols, and non-original reports were excluded from the synthesis. **Results:** The included studies comprised clinical biomarker studies, transcriptomic and receptor-expression analyses, experimental pulmonary hypertension models, chronic lung injury or fibrosis models, and pulmonary vascular cellular studies. Human evidence directly addressing chronic lung disease-associated pulmonary hypertension was limited and largely biomarker-centered, with CNP or NT-proCNP signals suggesting potential relevance to pulmonary vascular stress but without definitive NPR-C/NPR3 validation. Experimental studies supported biologic plausibility, linking CNP/NPR-C-related pathways to endothelial function, pulmonary vascular remodeling, smooth muscle and fibroblast behavior, hypoxic signaling, fibrosis, and right ventricular remodeling. However, findings varied across models, and receptor-specific attribution to NPR-C/NPR3 was often incomplete. **Conclusions:** Current evidence suggests that NPR-C/NPR3 is a biologically plausible but not yet clinically validated pathway in pulmonary hypertension associated with chronic lung disease. The strongest gap is the absence of prospective human studies integrating invasive hemodynamics, lung disease phenotyping, CNP/NT-proCNP profiling, NPR3 expression or genetic assessment, receptor-level biology, and right ventricular outcomes. Future studies should determine whether NPR-C/NPR3 can improve phenotyping, risk stratification, or therapeutic targeting in chronic lung disease-associated pulmonary hypertension.

## 1. Introduction

Pulmonary hypertension associated with chronic lung disease and/or hypoxia represents one of the most clinically heterogeneous forms of pulmonary hypertension. Within the contemporary clinical classification, this entity is categorized as Group 3 pulmonary hypertension and includes pulmonary vascular disease occurring in the context of chronic obstructive pulmonary disease, emphysema, interstitial lung disease, combined pulmonary fibrosis and emphysema, sleep-disordered breathing, hypoventilation syndromes, and other chronic hypoxic lung disorders. Pulmonary hypertension is currently defined hemodynamically by a mean pulmonary arterial pressure greater than 20 mmHg at rest. In the setting of suspected pre-capillary pulmonary vascular involvement, including pulmonary hypertension associated with chronic lung disease, the hemodynamic profile should also include a pulmonary arterial wedge pressure of 15 mmHg or less and a pulmonary vascular resistance greater than 2 Wood units [1]. In patients with parenchymal lung disease or chronic hypoxia, these hemodynamic thresholds require clinical interpretation alongside pulmonary vascular resistance, pulmonary arterial wedge pressure, lung mechanics, gas exchange, computed tomography findings, symptoms, exercise limitation, and right ventricular structure and function [1]. Patients with risk factors for post-capillary pulmonary hypertension or heart failure with preserved ejection fraction, including obesity, systemic hypertension, diabetes, atrial fibrillation, obstructive sleep apnea or hypoventilation, renal dysfunction, or echocardiographic evidence of diastolic dysfunction, may require careful assessment of left-heart contribution. In selected patients with borderline resting wedge pressure and clinical features suggesting occult post-capillary disease, exercise or fluid challenge during invasive hemodynamic assessment may help determine the dominant driver of pulmonary pressure elevation [1].

The diagnostic and management challenges in chronic lung disease-associated pulmonary hypertension arise from the overlap between parenchymal lung disease, pulmonary vascular remodeling, hypoxemia, inflammation, vascular pruning, and right ventricular adaptation. Dyspnea, exercise limitation, reduced diffusion capacity, oxygen desaturation, radiographic vascular changes, and right ventricular dysfunction may reflect lung disease severity, pulmonary vascular disease, cardiac comorbidity, or a combination of these processes. Current guidelines therefore recommend a multimodal diagnostic approach that integrates echocardiography, pulmonary function testing, arterial blood gases, computed tomography, and right heart catheterization when invasive confirmation is expected to influence management [1]. Management remains centered on optimization of the underlying lung disease, correction of hypoxemia and sleep-disordered breathing when present, pulmonary rehabilitation, expert-center referral for severe or uncertain cases, and selected pulmonary hypertension-directed therapy in carefully defined subgroups [1].

Despite advances in classification and clinical phenotyping, pulmonary hypertension associated with chronic lung disease remains difficult to stratify biologically. Natriuretic peptides, particularly B-type natriuretic peptide and N-terminal pro-B-type natriuretic peptide, are commonly used to assess cardiopulmonary stress and right ventricular strain, but they do not fully distinguish pulmonary vascular remodeling from parenchymal lung disease, left-heart disease, hypoxemia, inflammation, or renal dysfunction. This limitation has encouraged interest in pathways that may better connect endothelial biology, vascular remodeling, fibrosis, right ventricular adaptation, and biomarker interpretation. Among these, the C-type natriuretic peptide pathway and natriuretic peptide receptor-C, encoded by NPR3, represent an underexplored axis in pulmonary vascular disease.

NPR3 encodes natriuretic peptide receptor 3, commonly referred to as NPR-C. The receptor has historically been described as a clearance receptor responsible for removing circulating and extracellular natriuretic peptides through receptor-mediated endocytosis [2,3,4,5]. However, this functional description is incomplete. Experimental cardiovascular studies indicate that NPR-C can also mediate active signaling, including G-protein-linked effects relevant to vascular tone, endothelial integrity, cellular proliferation, inflammation, and remodeling [5]. In vascular biology, endothelial C-type natriuretic peptide signaling through NPR-C has been shown to contribute to vascular homeostasis, and small-molecule NPR-C agonism has been proposed as a potentially druggable approach in cardiovascular disease models [6].

The biological plausibility of NPR-C/NPR3 in chronic lung disease-associated pulmonary hypertension is strengthened by the central role of endothelial dysfunction, hypoxic vascular remodeling, smooth muscle proliferation, adventitial fibrosis, inflammatory signaling, and right ventricular remodeling in this condition. C-type natriuretic peptide is produced locally in vascular tissues and may act in an autocrine or paracrine manner, making it conceptually distinct from natriuretic peptides that primarily reflect myocardial wall stress [7]. In experimental pulmonary hypertension, C-type natriuretic peptide pathway modulation has produced mixed findings: some models support protective effects on pulmonary vascular remodeling, whereas others show no attenuation of pulmonary hypertension in chronic hypoxia or SU5416-hypoxia conditions [8]. This variability suggests that the relevance of C-type natriuretic peptide and NPR-C/NPR3 may depend on disease phenotype, timing, receptor expression, cell type, severity of vascular injury, and the balance between clearance and signaling functions.

Clinical evidence remains limited and largely ligand- or biomarker-centered. Recent data suggest that N-terminal pro-C-type natriuretic peptide may have diagnostic relevance in chronic obstructive pulmonary disease-associated pulmonary hypertension, but such biomarker findings do not establish whether NPR-C/NPR3 itself is altered, causal, protective, maladaptive, or clinically actionable [9]. Similarly, broader pulmonary hypertension and chronic lung disease literature has not yet clarified whether NPR-C/NPR3 can improve diagnostic discrimination, identify disproportionate pulmonary vascular disease, predict right ventricular maladaptation, or guide therapy beyond established clinical tools. This distinction is important because a circulating peptide signal may reflect endothelial activation, lung injury, right ventricular stress, altered peptide processing, receptor-mediated clearance, or several of these processes simultaneously.

A focused evidence map is therefore needed. Existing reviews of Group 3 pulmonary hypertension address epidemiology, pathobiology, diagnosis, and management, but they do not systematically map original empirical evidence on NPR-C/NPR3 as a receptor-level pathway in pulmonary hypertension and chronic lung disease [10]. Conversely, reviews of natriuretic peptide biology describe NPR-C signaling and vascular effects but do not specifically synthesize the evidence in chronic lung disease-associated pulmonary hypertension [5]. This creates a translational gap between molecular vascular biology and clinical cardiopulmonary phenotyping.

Accordingly, this PRISMA-ScR scoping review was undertaken to map original empirical evidence on NPR-C/NPR3 and related C-type natriuretic peptide biology in pulmonary hypertension associated with chronic lung disease. The review focuses on diagnosis, prognostic stratification, mechanistic phenotyping, and management relevance, while distinguishing direct human PH-CLD evidence from indirect pulmonary hypertension, animal-model, chronic lung injury, and cellular mechanistic evidence. By defining the extent and limits of the current evidence base, this review aims to clarify whether NPR-C/NPR3 is best viewed at present as a biomarker candidate, a mechanistic mediator, a therapeutic hypothesis, or a research gap requiring prospective translational validation.

## 2. Methods

### 2.1. Study Design and Reporting Standards

This scoping review was conducted to map original empirical evidence on natriuretic peptide receptor-C, encoded by NPR3, in pulmonary hypertension associated with chronic lung disease, with attention to diagnostic, prognostic, mechanistic, and management-related implications. The review was designed and reported in accordance with the Preferred Reporting Items for Systematic Reviews and Meta-Analyses extension for Scoping Reviews (PRISMA-ScR), with applicable PRISMA 2020 reporting elements addressed where relevant to a scoping review [11,12]. The completed PRISMA-ScR checklist is provided as Supplementary File S1, and the study-selection process is presented using a PRISMA flow diagram in Figure 1. The electronic search strategy was reported in accordance with PRISMA-S guidance for reproducible search reporting. The methodological structure was informed by Joanna Briggs Institute guidance for scoping reviews, using the Population–Concept–Context framework to define eligibility criteria and organize evidence synthesis [13].

Searches of the executed databases, PubMed/MEDLINE and APA PsycINFO, identified 72 database records, including 70 from PubMed/MEDLINE and 2 from APA PsycINFO. The Web of Science Core Collection strategy reported in Supplementary File S2 was developed for reproducibility but was not executed and contributed no records to this PRISMA flow diagram. After removal of 2 duplicate records, 70 records underwent title and abstract screening. Forty-two records were excluded, and 28 reports were sought for full-text retrieval. All 28 reports were retrieved and assessed for eligibility. Eleven full-text reports were excluded with reasons, leaving 17 original studies for inclusion in the scoping review.

A protocol was developed before screening. The protocol was not registered; however, eligibility criteria, search strategies, screening procedures, and data-charting procedures were defined before evidence selection. The review was not designed to estimate pooled treatment effects; rather, it was intended to define the extent, nature, and translational relevance of the available evidence.

### 2.2. Eligibility Criteria

Eligibility criteria were defined according to the Population–Concept–Context framework. The population included human studies involving confirmed or suspected pulmonary hypertension associated with chronic lung disease or hypoxia, including chronic obstructive pulmonary disease, emphysema, interstitial lung disease, pulmonary fibrosis, combined pulmonary fibrosis and emphysema, sleep-disordered breathing, hypoventilation syndromes, bronchopulmonary dysplasia, and other chronic hypoxic lung diseases. Experimental animal and cellular studies were eligible when they examined pulmonary hypertension, pulmonary vascular remodeling, chronic hypoxia, chronic lung injury, lung fibrosis, emphysema-related biology, right ventricular remodeling, or pulmonary vascular cell behavior relevant to chronic lung disease-associated pulmonary hypertension.

The concept of interest was NPR-C/NPR3 and receptor-level natriuretic peptide biology. Eligible studies directly assessed NPR-C, NPR3, natriuretic peptide receptor-C, natriuretic peptide receptor 3, NPR-C-mediated peptide clearance, NPR-C signaling, NPR3 genetic variation, receptor expression, receptor binding, downstream signaling, or C-type natriuretic peptide biology with a clear pulmonary vascular, chronic lung disease, or right ventricular context. Studies focused only on BNP, NT-proBNP, ANP, CNP, or NT-proCNP as nonspecific biomarkers were eligible only when the study provided a receptor-level interpretation or a clear mechanistic link to NPR-C/NPR3-related biology.

The context was diagnosis, phenotyping, prognosis, pathobiology, or management of pulmonary hypertension associated with chronic lung disease. Studies in pulmonary arterial hypertension or other pulmonary hypertension phenotypes were eligible only when they provided original receptor-level or pathway-level evidence directly relevant to pulmonary vascular remodeling, right ventricular adaptation, hypoxic remodeling, or natriuretic peptide receptor biology.

Only original empirical studies were included. Eligible designs included randomized and non-randomized interventional studies, cohort studies, case–control studies, cross-sectional studies, diagnostic studies, prognostic studies, genetic association studies, transcriptomic or proteomic studies, tissue-based studies, animal studies, and in vitro mechanistic studies. Case reports or case series were eligible only if they contained extractable primary data on NPR-C/NPR3 or receptor-level natriuretic peptide biology. Reviews, systematic reviews, meta-analyses, editorials, commentaries, guidelines, protocols, book chapters, letters without original data, and conference abstracts without sufficient extractable primary data were excluded from the evidence synthesis. Reviews were not excluded at the search stage because their reference lists could identify additional original studies.

### 2.3. Information Sources and Search Strategy

Electronic searches were conducted in PubMed/MEDLINE and APA PsycINFO via APA PsycNet from database inception to 18 May 2026. A Web of Science Core Collection search strategy was developed for reproducibility and is reported in Supplementary File S2; however, this database search was not executed and was not included in the final PRISMA counts. PubMed/MEDLINE was selected as the principal biomedical database because the review question focused on pulmonary vascular biology, chronic lung disease, pulmonary hypertension, and receptor-level natriuretic peptide biology. APA PsycINFO was included as a supplementary source because the Population–Concept–Context framework included sleep-disordered breathing, hypoventilation, dyspnea-related phenotyping, and behavioral or functional consequences of chronic cardiopulmonary disease. APA PsycINFO was not considered a primary vascular biology database, and its contribution to the final record set was limited. The Web of Science strategy was developed during search planning to support transparent reporting and possible future updating, but it was not executed before the final exported record set was locked. Therefore, no Web of Science records were screened, excluded, included, or counted in the PRISMA flow diagram.

The search strategy combined controlled vocabulary and free-text terms for three domains: NPR-C/NPR3 and C-type natriuretic peptide receptor biology; pulmonary hypertension, pulmonary vascular remodeling, pulmonary vascular resistance, and right ventricular remodeling; and chronic lung disease, hypoxia, COPD, emphysema, interstitial lung disease, pulmonary fibrosis, sleep-disordered breathing, hypoventilation, and relevant experimental lung injury models. PubMed/MEDLINE searches used MeSH terms and title/abstract terms. APA PsycINFO searches used the APA PsycNet Advanced Search interface. The Web of Science strategy was formatted using Topic Search syntax for reproducibility.

No date, language, publication-type, age-group, or species filters were applied at the search stage. Reviews and meta-analyses were retained during searching and initial record management to support citation tracking but were excluded during eligibility assessment if they did not contain original empirical data. The full reproducible search syntax for each database is provided in Supplementary File S2.

### 2.4. Record Management and Deduplication

Database records were exported in citation-management-compatible formats. PubMed/MEDLINE records were exported in .nbib format, and APA PsycINFO records were exported in RIS-compatible format. All records were imported into a single Zotero library. Source tags were applied before deduplication to preserve database provenance. Duplicate records were identified using Zotero’s duplicate-detection function and then manually checked by DOI, PMID where available, exact or near-exact title, first author, journal, and publication year. Duplicate records were merged rather than deleted to preserve source metadata, tags, and collection membership.

After deduplication, the unique record set was exported to the screening log. The number of records identified, duplicates removed, records screened, reports sought for retrieval, reports assessed for eligibility, full-text exclusions, and included studies was recorded for the PRISMA flow diagram.

### 2.5. Study Selection

Study selection occurred in two stages. First, titles and abstracts were screened against the eligibility criteria. Records were classified as include, exclude, or unclear. Records classified as include or unclear proceeded to full-text review. Second, full texts were assessed for eligibility using the same Population–Concept–Context framework.

Screening was performed independently by two reviewers (M.A. and N.I.). Disagreements were resolved by consensus, with adjudication by E.E.E. when required. Reasons for exclusion were recorded at the full-text stage using prespecified categories: non-original article, no direct NPR-C/NPR3 or receptor-level natriuretic peptide evidence, wrong population or disease context, biomarker-only study without NPR-C/NPR3-specific interpretation, duplicate publication or correction, insufficient extractable data, or conference abstract without adequate primary data.

### 2.6. Data Charting

Data were charted using a standardized extraction form developed before full-text assessment. The form was piloted on a sample of included studies and refined for completeness and consistency. Data charting was performed by one reviewer and verified by a second reviewer. Discrepancies were resolved through discussion.

Extracted variables included author, year, country, journal, study design, study setting, population or experimental model, sample size, disease context, pulmonary hypertension definition, chronic lung disease phenotype, NPR-C/NPR3-related exposure or measurement, natriuretic peptide pathway variables, comparator or control group where applicable, diagnostic outcomes, prognostic outcomes, hemodynamic outcomes, imaging outcomes, functional outcomes, mechanistic outcomes, right ventricular outcomes, management relevance, principal findings, and study limitations.

Included studies were classified by evidence domain as human clinical biomarker or phenotyping studies, human receptor-level or genetic signal studies, hypoxia or pulmonary vascular receptor-regulation studies, experimental pulmonary hypertension or right ventricular remodeling studies, chronic lung injury or fibrosis studies, and pulmonary vascular cellular or metabolic mechanism studies.

### 2.7. Evidence Synthesis

A descriptive and narrative synthesis was performed. Meta-analysis was not planned because the included studies were expected to differ substantially in design, population, disease phenotype, model system, NPR-C/NPR3 measurement approach, and outcome definition. Evidence was first summarized numerically by study type, disease context, evidence domain, and primary outcome category. Findings were then synthesized thematically according to diagnostic relevance, prognostic relevance, mechanistic evidence, therapeutic or management relevance, and translational gaps.

Evidence was interpreted according to degree of directness. Human studies in pulmonary hypertension associated with chronic lung disease were considered the most directly applicable. Human studies in other pulmonary hypertension phenotypes were interpreted as supportive but indirect when they addressed NPR-C/NPR3, CNP-pathway biology, pulmonary vascular remodeling, or right ventricular adaptation. Animal and cellular studies were used to map mechanistic plausibility and generate translational hypotheses, not to infer clinical effectiveness.

The synthesis distinguished evidence directly involving NPR-C/NPR3 from broader C-type natriuretic peptide or natriuretic peptide receptor pathway evidence. Where studies assessed CNP or NT-proCNP without direct receptor measurement, conclusions were framed as pathway-level or ligand-level evidence rather than receptor-specific evidence.

### 2.8. Critical Appraisal

Formal risk-of-bias appraisal was not used as an inclusion criterion because the purpose of the review was to map the scope and nature of the evidence rather than to estimate a pooled effect or determine intervention efficacy. However, methodological features relevant to interpretation were charted, including study design, sample size, phenotyping quality, use of right heart catheterization, chronic lung disease characterization, receptor-specific measurement, comparator selection, replication, and outcome ascertainment. These limitations were incorporated into the narrative synthesis and research-gap analysis.

### 2.9. Patient and Public Involvement

Patients or members of the public were not involved in the design, conduct, analysis, or reporting of this scoping review.

### 2.10. PRISMA Checklist and Flow Diagram

The completed PRISMA-ScR checklist is provided as Supplementary File S1. The PRISMA flow diagram is included in the main text as Figure 1. The complete electronic search strategies are provided in Supplementary File S2.

## 3. Results

### 3.1. Study Selection

The database search identified 72 records. After removal of 2 duplicate records, 70 unique records were screened by title and abstract. Of these, 42 records were excluded at title/abstract screening because they did not address NPR-C/NPR3, receptor-level natriuretic peptide biology, pulmonary hypertension, chronic lung disease, pulmonary vascular remodeling, or right ventricular remodeling. Twenty-eight reports were sought for full-text retrieval, and all were retrieved. After full-text eligibility assessment, 11 reports were excluded: 3 lacked direct NPR-C/NPR3 or receptor-level natriuretic peptide evidence, 3 were reviews, meta-analyses, editorials, commentaries, or other non-original articles, 2 had the wrong population or disease context, 2 reported natriuretic peptides only as nonspecific biomarkers without an NPR-C/NPR3-specific or receptor-level link, and 1 was a duplicate publication, erratum, correction, or report with insufficient extractable data. Seventeen original studies were included in the scoping review and evidence map. The study-selection process is summarized in Figure 1.

### 3.2. Characteristics of Included Studies

The included evidence base was heterogeneous and spanned clinical biomarker studies, pulmonary hypertension hemodynamic studies, animal models, lung fibrosis or injury models, pulmonary vascular cell studies, and transcriptomic or receptor-expression studies. The publication period ranged from early clinical and experimental natriuretic peptide studies in the 1990s to recent translational studies examining CNP, NPR-C/NPR3, NPR-B/GC-B, and pulmonary vascular remodeling in human and experimental pulmonary hypertension. Early human work measured circulating C-type natriuretic peptide in cor pulmonale and congestive heart failure [14], while later clinical studies assessed NT-proCNP or broader natriuretic peptide profiles in COPD-associated pulmonary hypertension and precapillary pulmonary hypertension [9,15].

By primary evidence domain, the included studies were grouped into six categories: human clinical biomarker/phenotyping studies, human receptor-level or genetic signal studies, hypoxia and pulmonary vascular receptor-regulation studies, experimental pulmonary hypertension or right ventricular remodeling studies, chronic lung injury or fibrosis studies, and pulmonary vascular cellular or metabolic mechanism studies. The distribution of included studies by primary evidence domain is presented in Table 2. The primary evidence-domain distribution is shown in Figure 2. No review articles or meta-analyses were included in the evidence map. The characteristics and primary findings of the included original studies are summarized in Table 1 [16,17,18,19,20]. Several studies were mechanistically relevant to NPR-C/NPR3 but did not focus exclusively on Group 3 pulmonary hypertension; these were retained when they provided original receptor-level or CNP-pathway evidence directly relevant to pulmonary vascular remodeling, right ventricular remodeling, hypoxia, COPD, or chronic lung disease biology.

Each included study was assigned to one dominant domain for descriptive mapping. The evidence base was distributed across human clinical biomarker or phenotyping studies, human receptor-level or genetic signal studies, hypoxia and pulmonary vascular receptor-regulation studies, experimental pulmonary hypertension or right ventricular remodeling studies, chronic lung injury or fibrosis studies, and pulmonary vascular cellular or metabolic mechanism studies.

Table 1 summarizes the included original empirical studies mapped in this scoping review. The ‘Evidence specificity’ column distinguishes direct NPR-C/NPR3 receptor or gene evidence from broader CNP-pathway evidence, CNP/NPR-B/GC-B pathway evidence, and ligand-only biomarker evidence without receptor-level assessment. Studies are organized by evidence domain, study type, disease or model context, NPR-C/NPR3-related exposure, principal outcome domain, key finding, and relevance to pulmonary hypertension associated with chronic lung disease. Reviews, meta-analyses, editorials, commentaries, and non-original reports were excluded from the evidence map. Abbreviations: CNP, C-type natriuretic peptide; COPD, chronic obstructive pulmonary disease; GC-B, guanylyl cyclase-B; ILD, interstitial lung disease; mPAP, mean pulmonary arterial pressure; NPR-C, natriuretic peptide receptor-C; NPR3, gene encoding natriuretic peptide receptor-C; NT-proCNP, N-terminal pro-C-type natriuretic peptide; PAH, pulmonary arterial hypertension; PH, pulmonary hypertension; PH-CLD, pulmonary hypertension associated with chronic lung disease; PVR, pulmonary vascular resistance; RV, right ventricle/right ventricular.

Table 2 shows the distribution of included original studies according to their primary evidence domain. Each study was assigned to one dominant category to avoid double counting, although several studies contributed evidence across more than one mechanistic or translational domain.

### 3.3. Human Clinical and Biomarker Evidence

Human evidence directly linking the NPR-C/NPR3 axis to pulmonary hypertension associated with chronic lung disease was limited. The most directly relevant contemporary clinical study assessed plasma NT-proCNP in COPD patients and divided participants into those without pulmonary hypertension and those with pulmonary hypertension using systolic pulmonary artery pressure thresholds [9]. In that study, COPD patients with pulmonary hypertension had higher NT-proCNP levels than those without pulmonary hypertension, and NT-proCNP correlated with systolic pulmonary artery pressure, dyspnea score, COPD Assessment Test score, oxygen saturation, C-reactive protein, and fibrinogen [9]. The same study reported an area under the receiver-operating characteristic curve of 0.82 for NT-proCNP in predicting pulmonary hypertension in COPD, with an estimated threshold of 2.16 pg/mL, sensitivity of 84.6%, and specificity of 81.0% [9].

Earlier clinical biomarker evidence suggested that circulating CNP may be increased in cor pulmonale, although the available study-level evidence was limited by small sample size and incomplete hemodynamic characterization [14]. A broader precapillary pulmonary hypertension biomarker study evaluated circulating natriuretic peptides in relation to hemodynamics, providing supportive evidence that CNP-related biomarkers may reflect cardiopulmonary vascular stress, but this evidence was not specific to chronic lung disease-associated pulmonary hypertension [15]. Across the clinical evidence base, no included human PH-CLD study simultaneously measured NPR3 expression, NPR-C protein abundance, receptor activity, circulating CNP/NT-proCNP, and right heart catheterization-confirmed pulmonary hemodynamics in the same cohort.

### 3.4. Genomic, Transcriptomic, and Receptor-Expression Evidence

Transcriptomic and receptor-expression studies provided biologic plausibility for NPR-C/NPR3 involvement in pulmonary vascular disease, but most were hypothesis-generating rather than clinically validated. In hypoxic pulmonary hypertension-related transcriptomic work, Npr3 emerged among candidate genes identified during analysis of pulmonary vascular disease progression, supporting the inclusion of NPR3 in the broader hypoxia-remodeling evidence map [28]. In human pulmonary hypertension transcriptomic work, NPR3 was reported among genes linked to epigenetically reactivated developmental programs, suggesting that NPR3 may participate in abnormal pulmonary vascular cellular states rather than functioning only as a passive natriuretic peptide clearance pathway [29].

Cellular receptor-expression studies also supported a mechanistic link between growth-factor signaling and reduced NPR-C expression. In lung arterial smooth muscle cells, fibroblast growth factor-1, fibroblast growth factor-2, and platelet-derived growth factor-BB induced dose- and time-dependent reductions in NPR-C mRNA, implicating tyrosine kinase growth-factor signaling as a mechanism through which hypoxia-responsive remodeling pathways may suppress NPR-C [21]. This finding is relevant to pulmonary hypertension associated with chronic lung disease because hypoxia, growth-factor activation, and pulmonary artery smooth muscle proliferation are central features of hypoxic pulmonary vascular remodeling.

### 3.5. Experimental Pulmonary Hypertension Models

Experimental pulmonary hypertension studies produced directionally mixed but informative findings. In a monocrotaline rat model, C-type natriuretic peptide was reported to ameliorate pulmonary hypertension, supporting the concept that CNP-pathway activation may reduce pulmonary vascular remodeling in selected inflammatory or toxic vascular injury models [22]. In contrast, a later study using chronic hypoxia and SU5416 plus hypoxia models found that continuous intravenous CNP infusion did not attenuate pulmonary hypertension, right ventricular hypertrophy, or vascular remodeling, despite increasing serum CNP levels [8]. This discordance indicates that the biological effect of CNP-pathway modulation may depend strongly on the experimental model, timing, route of delivery, receptor context, and severity of endothelial injury. Importantly, the negative study by Casserly et al. used continuous CNP ligand infusion rather than direct NPR-C/NPR3 receptor manipulation [8]. It should therefore be interpreted as evidence that exogenous CNP infusion did not modify pulmonary hypertension in those experimental contexts, not as definitive evidence against receptor-level NPR-C/NPR3 relevance.

More recent work strengthened the mechanistic case for endogenous endothelial CNP signaling in pulmonary hypertension. In SuHx-exposed mice, endothelial-restricted CNP deletion accentuated pulmonary hypertension, right ventricular hypertrophy, and right ventricular fibrosis, while global NPR-C deletion specifically worsened right ventricular hypertrophy and fibrosis without substantially altering right ventricular systolic pressure [8]. The same study found that exogenous CNP reduced right ventricular systolic pressure and promoted anti-proliferative and anti-remodeling signaling, providing direct evidence that CNP-dependent pathways involving NPR-B and NPR-C may influence both pulmonary vascular disease and right ventricular remodeling [8].

Additional experimental evidence emphasized the importance of receptor context. A constitutively active form of natriuretic peptide receptor 2 was reported to ameliorate experimental pulmonary arterial hypertension, indicating that sustained CNP/NPR2/GC-B pathway activation can modify pulmonary vascular disease in experimental systems [8,30]. Endothelial CNP/GC-B signaling also prevented pulmonary hypertension progression in monocrotaline-pyrrole and hypoxia models, and endothelial-cell-specific loss of CNP or GC-B worsened pulmonary vascular and right ventricular phenotypes [27]. Although this latter evidence primarily implicates NPR-B/GC-B rather than NPR-C, it reinforces the broader conclusion that CNP-receptor biology is active within pulmonary vascular disease rather than merely reflecting biomarker leakage from injured tissue [27].

### 3.6. Chronic Lung Disease, Fibrosis, and Lung Injury Models

The chronic lung disease-adjacent evidence was strongest for pulmonary fibrosis and lung injury models rather than COPD-associated pulmonary hypertension models. In a bleomycin-induced lung fibrosis model, continuous CNP infusion attenuated pulmonary fibrosis, and fibroblast-targeted CNP expression suppressed fibrotic changes compared with wild-type littermates [31]. The same study concluded that CNP exerted anti-inflammatory and anti-fibrotic effects through lung fibroblasts, possibly by disturbing TGF-β–Smad signaling and attenuating fibroblast-to-myofibroblast transformation [31]. These findings are relevant to pulmonary hypertension associated with interstitial lung disease because pulmonary fibrosis, fibroblast activation, extracellular matrix deposition, and secondary vascular remodeling frequently coexist in chronic parenchymal lung disease.

Additional included lung injury and fibrosis studies suggested that CNP/NPR-C-related biology may extend beyond pulmonary vascular tone into inflammatory and fibrotic lung remodeling [31]. However, the available studies did not establish whether these anti-inflammatory or anti-fibrotic effects were mediated through NPR-C/NPR3, NPR-B/GC-B, or mixed receptor-dependent mechanisms. This distinction is important because the scoping review question focuses on NPR-C/NPR3, while much of the pulmonary fibrosis evidence remains ligand-centered rather than receptor-specific.

### 3.7. Pulmonary Vascular Cellular Mechanisms

Cellular studies suggested that CNP-pathway signaling may oppose pulmonary vascular remodeling through anti-proliferative, anti-migratory, anti-fibrotic, and metabolic effects. In PAH lung pericytes, CNP/GC-B/cGMP signaling reduced growth factor-induced proliferation, migration, and smooth muscle-like transdifferentiation through cGMP-dependent protein kinase I, inhibition of PI3K/AKT signaling, and activation of FoxO3 [24]. A later pericyte study found that PAH pericytes displayed increased glucose uptake, glycolysis, and de novo pyrimidine synthesis, and that CNP counteracted these metabolic changes through cGMP-dependent mechanisms involving reduced HIF-1α and GLUT-1 expression and inhibition of CAD activation [25].

These cellular findings support a remodeling-focused role for CNP receptor biology, but they do not by themselves prove that NPR-C/NPR3 is the dominant receptor in pulmonary vascular remodeling. In fact, the pericyte studies primarily emphasized GC-B/cGMP signaling, while NPR-C was discussed as a receptor that may generate distinct and cell-specific signaling responses [24]. The most direct NPR-C cellular evidence came from lung arterial smooth muscle cell studies showing growth-factor-mediated suppression of NPR-C mRNA, which links NPR-C expression to the proliferative signaling environment characteristic of hypoxic pulmonary vascular remodeling.

### 3.8. Synthesis of Key Findings

Across the 17 included original studies, four findings emerged. First, human clinical evidence in chronic lung disease-associated pulmonary hypertension remains biomarker-centered, with NT-proCNP showing promising diagnostic associations in COPD-associated pulmonary hypertension but without direct receptor-level NPR-C/NPR3 validation [9]. Second, transcriptomic and cellular studies support NPR3/NPR-C as a biologically plausible participant in pulmonary vascular remodeling, particularly in relation to hypoxia-responsive and growth-factor-driven pathways [28]. Third, animal studies show that CNP-pathway modulation can reduce pulmonary vascular or right ventricular remodeling in some models, but negative results in chronic hypoxia and SU5416 plus hypoxia models demonstrate that this effect is not uniform across pulmonary hypertension phenotypes [8]. Fourth, chronic lung disease-adjacent models suggest anti-inflammatory and anti-fibrotic effects of CNP in lung tissue, but the receptor-specific contribution of NPR-C/NPR3 remains incompletely resolved [31].

Overall, the evidence map indicates that NPR-C/NPR3 is a plausible but not yet clinically validated diagnostic, prognostic, or therapeutic target in pulmonary hypertension associated with chronic lung disease. The most immediate translational gap is the absence of prospective human PH-CLD cohorts that integrate right heart catheterization, detailed lung disease phenotyping, CNP/NT-proCNP measurement, NPR3 genetic or transcriptomic assessment, NPR-C protein or receptor-function assays, and right ventricular outcomes.

## 4. Discussion

### 4.1. Principal Findings from the Scoping Evidence Map

This scoping review mapped original empirical evidence on NPR-C/NPR3 and related C-type natriuretic peptide biology in pulmonary hypertension, chronic lung disease, pulmonary vascular remodeling, and right ventricular remodeling. The principal finding is that the evidence base is biologically plausible but clinically immature. Across the included original studies, NPR-C/NPR3 emerged less as an established diagnostic or therapeutic target and more as a plausible translational node connecting endothelial peptide signaling, natriuretic peptide clearance, pulmonary vascular remodeling, fibrotic lung injury, and right ventricular adaptation. The translational implications of the evidence map are summarized in Table 3.

Table 3 maps the current evidence base onto potential translational applications, including diagnosis, prognostic stratification, mechanistic phenotyping, therapeutic targeting, and management integration. The table distinguishes evidence that is directly supported by included original studies from areas that remain inferential or hypothesis-generating.

The most direct human evidence in chronic lung disease-associated pulmonary hypertension remains biomarker-based rather than receptor-based. Contemporary data suggest that NT-proCNP may be increased in COPD patients with pulmonary hypertension and may correlate with pulmonary pressure estimates and symptom burden, but these findings do not yet establish that NPR-C/NPR3 itself is causally involved or clinically actionable [9]. Earlier human work also reported increased circulating CNP in cor pulmonale, supporting the long-standing hypothesis that CNP biology is activated in cardiopulmonary vascular stress, although that evidence was limited by small sample size and older phenotyping methods [14].

The experimental evidence was more mechanistically informative but not uniform. In a monocrotaline rat model, continuous CNP administration attenuated pulmonary hypertension and vascular remodeling, suggesting a protective effect in a toxic-inflammatory pulmonary vascular injury model [22]. In contrast, CNP did not prevent pulmonary hypertension in chronic hypoxia or SU5416 plus hypoxia models, showing that CNP-pathway modulation does not produce consistent benefit across experimental pulmonary hypertension phenotypes [8]. More recent work has strengthened the case for endothelial CNP and natriuretic peptide receptor signaling in pulmonary vascular and right ventricular remodeling, but these data remain largely preclinical [26].

The chronic lung disease-adjacent evidence was strongest in pulmonary fibrosis and lung injury models. CNP attenuated bleomycin-induced pulmonary fibrosis in early experimental work, and later studies suggested that CNP may act on lung fibroblasts to reduce inflammatory and fibrotic remodeling [23]. These findings are relevant to pulmonary hypertension associated with interstitial lung disease, but they do not prove receptor-specific NPR-C/NPR3 mediation. From a clinical phenotyping perspective, ILD-associated pulmonary hypertension also extends beyond idiopathic fibrotic lung disease. Recent clinical work in primary Sjögren’s syndrome examined the relationship between pulmonary hypertension and interstitial lung disease, illustrating that autoimmune lung involvement, parenchymal disease, cardiovascular comorbidity, and pulmonary vascular assessment may intersect in ILD-PH phenotypes [32]. Although that study did not assess NPR-C/NPR3 and was therefore not part of the present evidence map, it supports the need for future PH-CLD studies to pre-specify ILD subtypes, including autoimmune ILD, rather than treating ILD-PH as a single homogeneous category. The evidence map therefore supports a cautious conclusion: CNP/NPR-C biology is relevant to several processes implicated in Group 3 pulmonary hypertension, but direct human evidence for NPR-C/NPR3 as a diagnostic, prognostic, or therapeutic target remains insufficient.

### 4.2. Interpretation in Context of Prior Syntheses

Prior syntheses of Group 3 pulmonary hypertension emphasize that pulmonary hypertension associated with chronic lung disease is not a single disease entity but a heterogeneous syndrome shaped by hypoxemia, parenchymal destruction, vascular pruning, inflammation, fibrosis, pulmonary vascular remodeling, and right ventricular dysfunction [10]. Current ESC/ERS guidance frames management around accurate phenotyping, interpretation of echocardiography alongside pulmonary function testing, gas exchange, diffusion capacity, and computed tomography, with right heart catheterization recommended when results are expected to influence management [1]. The same guideline framework emphasizes optimization of underlying lung disease, oxygenation, sleep-disordered breathing or hypoventilation where present, pulmonary rehabilitation, expert referral, transplant evaluation when appropriate, and selected use of PH-directed therapy in carefully defined subgroups [1].

Within that clinical framework, the present evidence map identifies a molecular gap. Natriuretic peptides are widely used as markers of cardiopulmonary stress, yet prior Group 3 PH syntheses do not substantially integrate receptor-level natriuretic peptide biology into diagnostic or management pathways. This matters because NPR-C has historically been described as a clearance receptor, but prior cardiovascular syntheses and experimental work support a more complex role involving Gi-linked signaling, adenylyl cyclase inhibition, phospholipase C activation, vascular tone, endothelial integrity, and remodeling biology [2,3,4,33]. Endothelial CNP/NPR-C signaling has also been shown to maintain vascular homeostasis and to represent a potentially druggable vascular pathway, expanding the conceptual role of NPR-C beyond peptide removal [6].

The present scoping map therefore extends prior syntheses in two ways. First, it shows that the CNP/NPR-C axis intersects with several established Group 3 PH mechanisms, including hypoxic remodeling, endothelial dysfunction, smooth muscle proliferation, fibrosis, and right ventricular remodeling. Second, it shows that the current literature has not yet translated this biology into clinically validated phenotyping. The evidence is sufficient to justify prospective translational studies, but it is not sufficient to recommend NPR-C/NPR3 measurement for routine diagnosis or management of pulmonary hypertension associated with chronic lung disease. An important implication of Group 3 PH heterogeneity is that the NPR-C/NPR3 working hypothesis is not equally supported across all chronic lung disease phenotypes. At present, the most disease-proximate support comes from COPD-associated PH or cor pulmonale, chronic hypoxic pulmonary vascular remodeling, and ILD/fibrosis-adjacent biology. Human biomarker data in cor pulmonale and COPD-PH suggest activation of the CNP/NT-proCNP axis [9,14], while hypoxia-adapted pulmonary vascular models and lung arterial smooth muscle studies support biological links between chronic hypoxia, growth-factor signaling, and altered NPR-C/NPR3-related receptor biology [16,17,21]. ILD and fibrotic lung disease are also plausible contexts because CNP-related pathways have been implicated in bleomycin-induced pulmonary fibrosis and fibroblast-associated remodeling [23,31]. However, this remains largely ligand-level or pathway-level evidence, and direct human NPR-C/NPR3 receptor validation in IPF-PH, autoimmune ILD-PH, or other fibrotic ILD-PH cohorts is lacking.

Conversely, several Group 3 PH contexts remain minimally represented or not represented in the current NPR-C/NPR3 evidence base. These include combined pulmonary fibrosis and emphysema, hypoventilation syndromes, isolated sleep-disordered breathing without daytime hypoventilation, high-altitude hypoxia, bronchopulmonary dysplasia, developmental lung disorders, and several mixed restrictive-obstructive phenotypes. The present evidence map therefore cannot determine whether the working hypothesis is more likely to be confirmed or refuted in these subgroups. The negative CNP-infusion findings in chronic hypoxia and SU5416-plus-hypoxia models [8] also argue for caution: they show that ligand-level pathway activation is not uniformly protective across pulmonary hypertension models, but they do not exclude cell-specific, receptor-specific, or disease-stage-specific roles for NPR-C/NPR3. Future studies should therefore stratify PH-CLD cohorts by COPD/emphysema, ILD/IPF, autoimmune ILD, CPFE, hypoventilation, and other Group 3 phenotypes before drawing disease-specific conclusions.

### 4.3. Gaps in the Literature and Future Directions

The most important gap is the lack of prospective human studies in well-characterized PH-CLD cohorts. The main evidence gaps and proposed approaches for addressing them are outlined in Table 4. Existing human evidence is largely peptide-centered, often relying on CNP or NT-proCNP measurements without simultaneous assessment of NPR3 expression, NPR-C protein abundance, receptor activity, lung disease phenotype, right ventricular imaging, and invasive hemodynamics. This creates uncertainty about whether altered CNP or NT-proCNP reflects pulmonary vascular disease, right ventricular strain, systemic inflammation, lung parenchymal injury, peptide clearance, receptor remodeling, or a combination of these processes.

Table 4 summarizes the principal evidence gaps identified by the scoping review and proposes corresponding research approaches. The gaps include limited human PH-CLD data, incomplete receptor-specific attribution, limited model-to-disease translation, absence of outcome-linked validation, and lack of integration into clinical management pathways.

A second gap is receptor specificity. Many studies implicate CNP biology, but CNP signals through more than one receptor pathway, particularly NPR-B/GC-B and NPR-C. Contemporary vascular biology supports distinct but overlapping roles for these receptors, and CNP can engage both cyclic GMP-dependent and NPR-C-associated signaling mechanisms depending on tissue, cell type, and disease context [34]. Without receptor-specific assays, genetic models, pharmacologic dissection, or cell-specific receptor mapping, it remains difficult to determine whether observed effects are attributable to NPR-C/NPR3, NPR-B/GC-B, or broader natriuretic peptide pathway activation.

A third gap is model-to-disease translation. Monocrotaline, chronic hypoxia, SU5416-hypoxia, bleomycin fibrosis, and cell culture systems each capture selected aspects of pulmonary vascular or lung injury biology, but none fully recapitulates the clinical complexity of COPD-PH, ILD-PH, combined pulmonary fibrosis and emphysema, or sleep-disordered breathing-associated PH. The discrepant CNP findings between monocrotaline and hypoxia/SU5416 models illustrate this problem clearly: a pathway that appears protective in one model may not be protective in another [22].

A fourth gap is the absence of outcome-linked biomarker validation. NT-proCNP may have diagnostic promise in COPD-associated pulmonary hypertension, but the evidence has not yet established whether it improves clinical decision-making beyond existing tools such as echocardiography, NT-proBNP, diffusion capacity, CT vascular markers, exercise testing, or right heart catheterization [9]. For NPR-C/NPR3 to become clinically useful, future studies must show whether receptor-level or ligand-level measurements add discrimination, calibration, prognostic value, or treatment-response information beyond standard clinical variables.

### 4.4. Conceptual Framework

A useful conceptual framework is to view NPR-C/NPR3 as a potential interface between pulmonary vascular stress, natriuretic peptide signaling, and right ventricular adaptation, rather than as a single isolated biomarker. In chronic lung disease, hypoxemia, parenchymal destruction, inflammation, fibrosis, and vascular pruning may induce endothelial dysfunction and pulmonary vascular remodeling. These processes may alter CNP release, NPR3 expression, NPR-C-mediated peptide clearance, and receptor signaling in endothelial cells, smooth muscle cells, fibroblasts, inflammatory cells, and right ventricular tissue.

In this model, circulating CNP or NT-proCNP represents only one visible layer of a deeper biological system. A rise in NT-proCNP could reflect endothelial activation, pulmonary vascular remodeling, inflammatory-fibrotic lung injury, impaired peptide processing, altered clearance, or right ventricular stress. NPR-C/NPR3 could influence this system through two non-exclusive mechanisms: by regulating local peptide availability through clearance and by participating in cell-specific signaling that affects vascular tone, inflammation, proliferation, fibrosis, or cardiac remodeling. This framework helps reconcile why ligand-centered studies may show signals of disease activity while receptor-level clinical translation remains incomplete. A proposed hypothesis-generating conceptual framework is presented in Figure 3.

NPR-C/NPR3 is presented as a hypothesis-generating interface between chronic lung disease, pulmonary vascular stress, natriuretic peptide signaling, disease phenotype, and clinical translation. The framework distinguishes evidence-supported domains, including ligand and biomarker signals, hypoxia and remodeling biology, and preclinical right ventricular effects, from unresolved domains, including receptor specificity, human NPR3/NPR-C assays, and clinical utility in PH-CLD.

The framework also clarifies why NPR-C/NPR3 should not yet be positioned as a stand-alone target. The available evidence instead supports a staged translational model: first, define NPR3/NPR-C expression and function across PH-CLD phenotypes; second, determine whether CNP/NT-proCNP and receptor-level measures map to invasive hemodynamics and right ventricular structure; third, test whether these measures predict clinical worsening or treatment response; and only then evaluate whether pathway modulation has therapeutic relevance.

### 4.5. Research Agenda

The immediate research priority is a prospective, multicenter PH-CLD cohort with rigorous phenotyping. A proposed minimum dataset for future prospective PH-CLD NPR-C/NPR3 studies is shown in Table 5. Clinically, future validation should evaluate NPR-C/NPR3 as an adjunct to established PH-CLD assessment rather than as a replacement for current diagnostic pathways. A practical translational design would integrate CNP/NT-proCNP and NPR3/NPR-C measures with echocardiographic PH probability, right ventricular size and function, right ventricular strain, right ventricular–pulmonary arterial coupling, and right heart catheterization-derived mPAP, PAWP, PVR, right atrial pressure, cardiac output, and cardiac index. These cardiovascular measures should be interpreted alongside lung disease phenotyping, including COPD/emphysema severity, ILD subtype, CPFE status, sleep-related hypoventilation, oxygen requirement, DLCO, arterial blood gases, CT parenchymal extent, pulmonary artery/aorta ratio, and exercise capacity. Such studies should test whether NPR-C/NPR3-related measures improve identification of a pulmonary vascular phenotype, predict right ventricular maladaptation, refine referral thresholds for expert PH evaluation, or add prognostic value beyond NT-proBNP, echocardiography, CT, pulmonary function testing, and invasive hemodynamics. Building on this framework, the cohort should intentionally include major PH-CLD phenotypes, including COPD, ILD, combined pulmonary fibrosis and emphysema, sleep-disordered breathing or hypoventilation syndromes, and chronic hypoxic lung disease, with pulmonary hypertension confirmed or excluded by right heart catheterization when clinically indicated.

Table 5 proposes a minimum dataset for future prospective studies evaluating NPR-C/NPR3 in pulmonary hypertension associated with chronic lung disease. The framework integrates clinical phenotyping, invasive hemodynamics, pulmonary function, imaging, circulating biomarkers, receptor-level biology, functional outcomes, and longitudinal events.

A second priority is receptor-specific mechanistic work in disease-relevant cells and tissues. Studies should distinguish NPR-C/NPR3 from NPR-B/GC-B signaling in pulmonary endothelial cells, pulmonary artery smooth muscle cells, adventitial fibroblasts, lung fibroblasts, inflammatory cells, and right ventricular fibroblasts or cardiomyocytes. This will require cell-specific expression mapping, loss- and gain-of-function models, receptor-selective pharmacology, ligand-clearance assays, and downstream signaling readouts.

A third priority is integration with management pathways. Future studies should test whether CNP/NT-proCNP and NPR-C/NPR3 measures identify patients with disproportionate pulmonary vascular disease, predict right ventricular maladaptation, or refine referral thresholds for expert PH evaluation. This direction is particularly important because current Group 3 PH management remains phenotype-driven and selective, with expert-center assessment recommended when pulmonary hypertension appears severe, uncertain, or management-changing [1].

Finally, therapeutic studies should proceed only after stronger mechanistic and clinical validation. The existing preclinical evidence supports biological plausibility but not therapeutic readiness. Future intervention studies should define the target population, receptor mechanism, timing of intervention, route of delivery, and safety implications before testing NPR-C/NPR3 modulation in humans. At present, NPR-C/NPR3 is best viewed as a promising translational hypothesis: a pathway that may help explain pulmonary vascular remodeling and right ventricular adaptation in chronic lung disease, but one that still requires disciplined validation before clinical implementation.

### 4.6. Limitations

Several limitations should be considered. First, the search strategy and PRISMA counts reflect only the databases that were actually searched and exported, namely PubMed/MEDLINE and APA PsycINFO. A Web of Science Core Collection strategy was developed and reported for reproducibility, but it was not executed and contributed no records to screening, eligibility assessment, or the PRISMA flow diagram. Embase and Scopus were also not searched. Because these databases may index biomedical, pharmacologic, conference, or non-MEDLINE records relevant to pulmonary vascular biology, the possibility of missed studies cannot be excluded. APA PsycINFO was used only as a supplementary database because the broader Population–Concept–Context framework included sleep-disordered breathing, hypoventilation, dyspnea-related phenotyping, and functional consequences of chronic cardiopulmonary disease; it should not be interpreted as a substitute for vascular biology-focused databases.

Second, the evidence base was uneven across Group 3 PH phenotypes. COPD-associated PH or cor pulmonale, chronic hypoxic remodeling, and ILD/fibrosis-adjacent biology were the best represented disease contexts, whereas CPFE, hypoventilation syndromes, high-altitude hypoxia, developmental lung disorders, bronchopulmonary dysplasia, and several mixed restrictive-obstructive phenotypes had little or no direct NPR-C/NPR3 evidence. Therefore, the review cannot determine whether the working hypothesis is equally applicable across all Group 3 PH subtypes.

Third, although the review was restricted to original empirical studies, much of the evidence was indirect for pulmonary hypertension associated with chronic lung disease and derived from pulmonary arterial hypertension, experimental pulmonary hypertension, chronic lung injury, fibrosis, or cellular models. Fourth, many included studies evaluated CNP or NT-proCNP without direct receptor-level assessment of NPR-C/NPR3, limiting receptor-specific interpretation. Fifth, data charting was performed by one reviewer and verified by a second reviewer rather than extracted independently in duplicate, which may increase the risk of extraction bias despite verification. Sixth, formal risk-of-bias assessment was not performed, consistent with the mapping purpose of a scoping review, but this limits conclusions about study quality and causal inference. Finally, heterogeneity in populations, models, exposures, receptor assays, and outcomes precluded quantitative synthesis.

## 5. Conclusions

This scoping review found that NPR-C/NPR3 and related C-type natriuretic peptide biology are biologically relevant to several mechanisms implicated in pulmonary hypertension associated with chronic lung disease, including endothelial dysfunction, pulmonary vascular remodeling, hypoxic signaling, fibrosis, inflammation, and right ventricular remodeling. However, the available evidence remains mainly preclinical or biomarker-based, and direct human data in well-characterized chronic lung disease-associated pulmonary hypertension cohorts are limited.

Current evidence does not support routine clinical use of NPR-C/NPR3 as a diagnostic biomarker, prognostic marker, or therapeutic target in this setting. The main gap is the absence of prospective studies integrating right heart catheterization, detailed lung disease phenotyping, CNP/NT-proCNP measurement, NPR3 genetic or transcriptomic assessment, receptor-level assays, right ventricular imaging, and longitudinal outcomes. Future research should clarify whether NPR-C/NPR3 improves disease phenotyping, risk stratification, or treatment-response assessment in pulmonary hypertension associated with chronic lung disease.

## Figures and Tables

**Figure 1:**
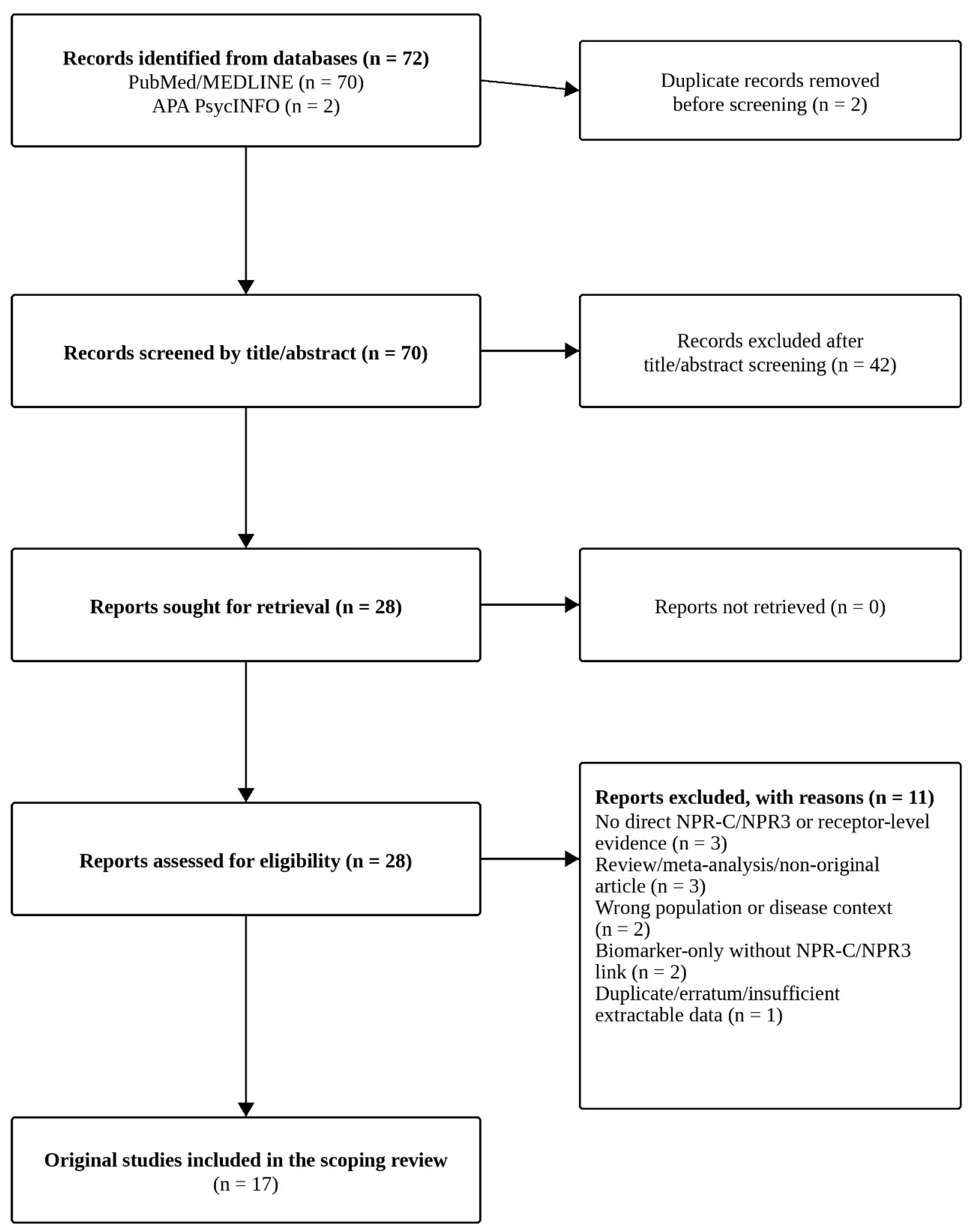
PRISMA flow diagram for study selection.

**Figure 2:**
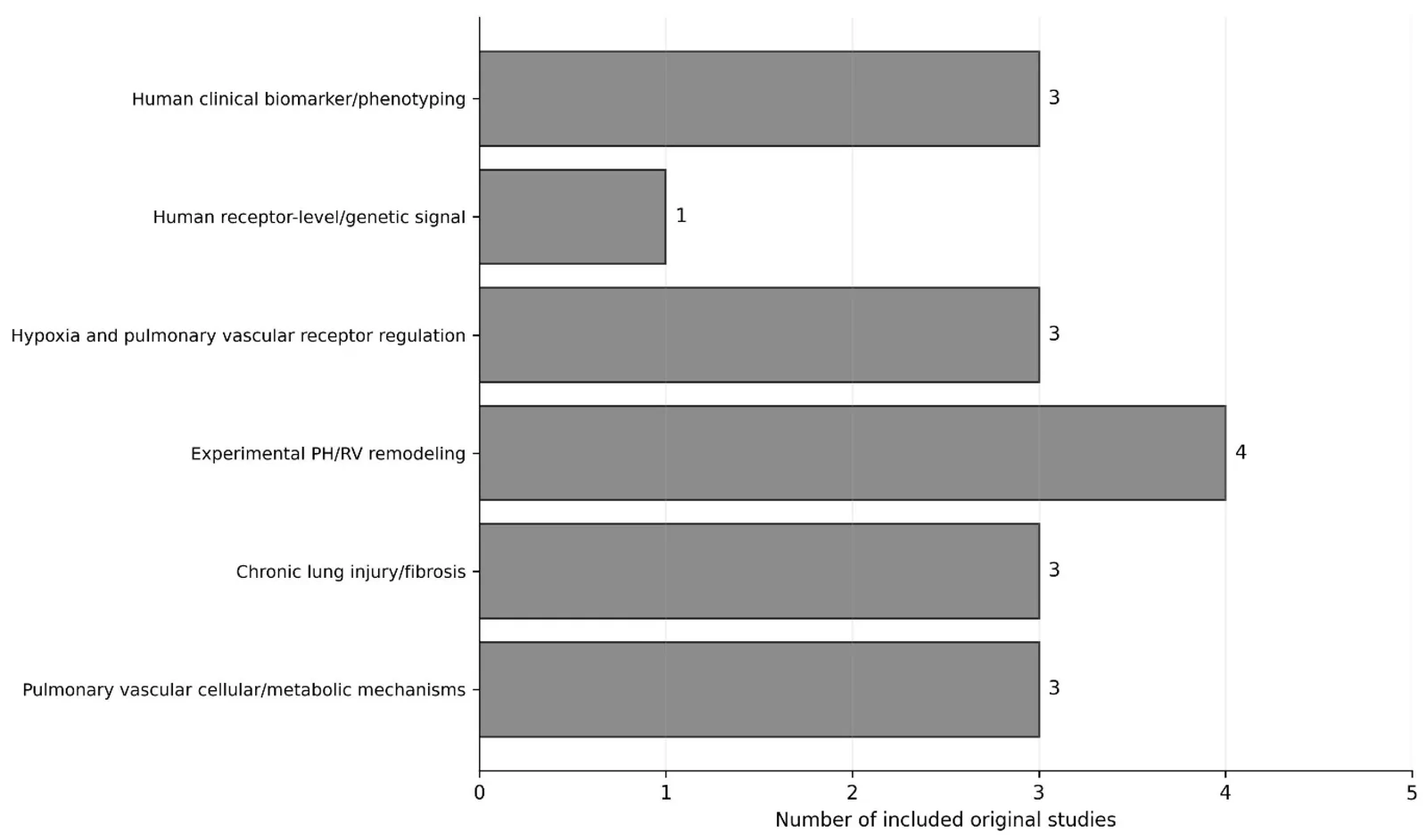
Distribution of included original studies by evidence domain.

**Figure 3:**
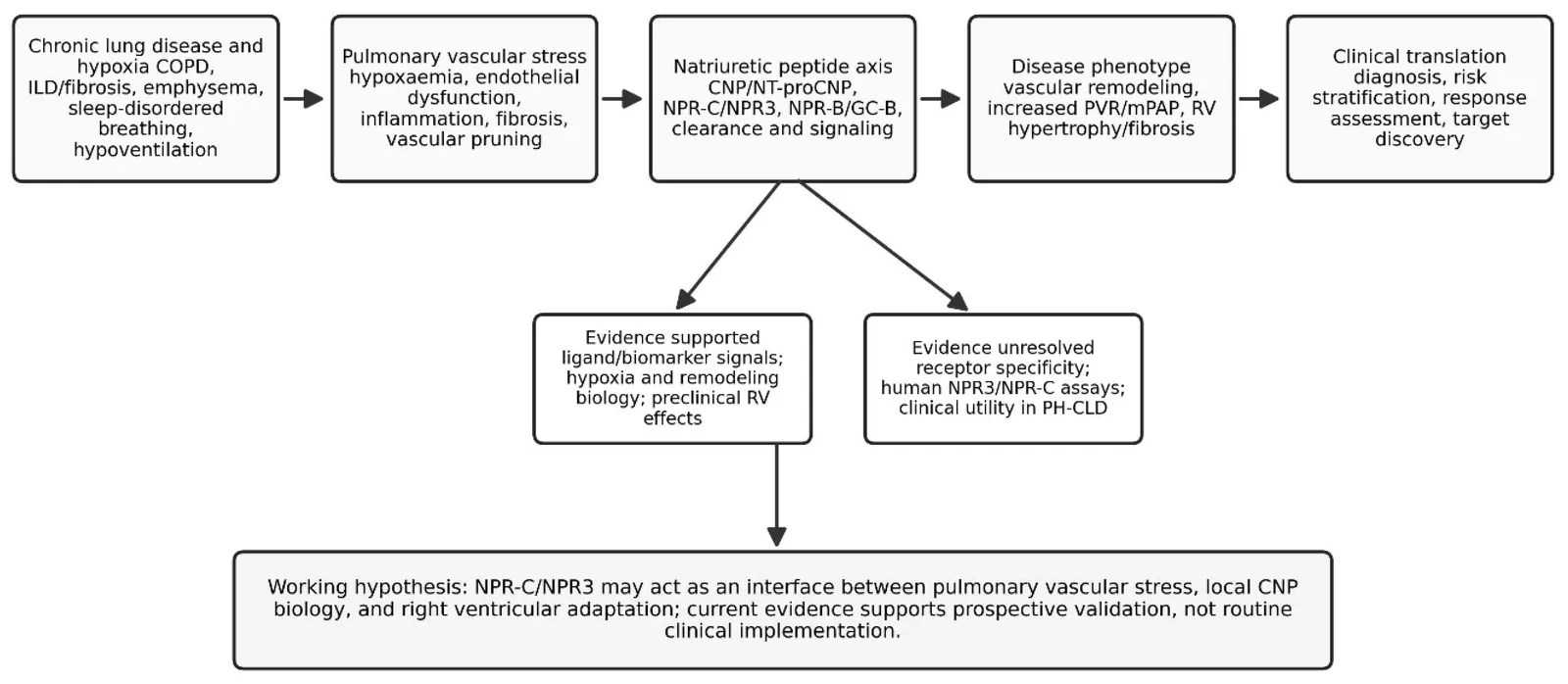
Conceptual framework for NPR-C/NPR3 in pulmonary hypertension associated with chronic lung disease.

**Table 1:** Characteristics and evidence map of included original studies.

Primary Evidence Domain	Study	Design	Population/Model	NPR-C/NPR3 or CNP Construct	Evidence Specificity	Key Finding	Interpretation for PH-CLD
Human clinical biomarker/PH-CLD	Cargill et al. 1994 [14]	Cross-sectional clinical biomarker study	Hypoxaemic cor pulmonale, congestive heart failure, and control groups	Plasma C-type natriuretic peptide (CNP)	Ligand-only human biomarker evidence; CNP measured, no NPR-C/NPR3 assay	CNP was increased in hypoxaemic cor pulmonale compared with age-matched controls; invasive pulmonary haemodynamics were not reported.	Direct chronic lung disease/cor pulmonale signal; receptor-level NPR-C/NPR3 was not measured.
Hypoxia-adapted pulmonary vascular model	Klinger et al. 1998 [17]	Experimental rat study	Normoxic and hypoxia-adapted rats	Pulmonary CNP expression and vasodilator response	CNP expression/vasodilator pathway evidence; not direct NPR-C/NPR3 manipulation	CNP expression and pulmonary vasodilator responses were evaluated in hypoxia-adapted rats, supporting altered CNP responsiveness in chronic hypoxia.	Relevant to hypoxia-associated pulmonary vascular disease in Group 3 PH.
NPR-C/NPR3 hypoxia regulation	Li et al. 1995 [16]	Experimental hypoxia/receptor-expression study	ANP knockout mice exposed to chronic hypoxia	Atrial natriuretic peptide clearance receptor/NPR-C gene expression	Direct NPR-C/NPR3-related hypoxia receptor-expression evidence	Hypoxia reduced NPR-C gene expression, consistent with transcriptional downregulation of the clearance receptor pathway.	Direct Npr3/NPR-C evidence in chronic hypoxic lung vascular context.
Pulmonary artery smooth muscle receptor regulation	Sun et al. 2001 [21]	In vitro lung arterial smooth muscle study	Cultured lung arterial smooth muscle cells	NPR-C mRNA after FGF and PDGF stimulation	Direct NPR-C mRNA regulation in pulmonary arterial smooth muscle cells	Tyrosine kinase receptor activation inhibited NPR-C expression in lung arterial smooth muscle cells and implicated mitogen-activated signaling pathways.	Links hypoxia-responsive growth factors and vascular remodeling to loss of NPR-C expression.
Experimental pulmonary hypertension	Itoh et al. 2004 [22]	Monocrotaline rat pulmonary hypertension model	Rats with monocrotaline-induced pulmonary hypertension	Continuous CNP infusion	CNP ligand-infusion evidence; receptor attribution unresolved	CNP ameliorated monocrotaline-induced pulmonary hypertension and pulmonary vascular remodeling.	Protective signal in a toxic-inflammatory PH model; receptor-specific NPR-C attribution remained incomplete.
Fibrotic lung disease model	Murakami et al. 2004 [23]	Bleomycin-induced pulmonary fibrosis model	Mice with bleomycin-induced lung fibrosis	Continuous CNP infusion	CNP ligand-infusion fibrosis evidence; receptor attribution unresolved	CNP attenuated bleomycin-induced pulmonary fibrosis.	Interstitial lung disease-adjacent evidence for anti-fibrotic CNP biology; NPR-C specificity not established.
Experimental pulmonary hypertension	Casserly et al. 2011 [8]	Chronic hypoxia and SU5416-plus-hypoxia rat models	Rats with hypoxic pulmonary hypertension and VEGF-receptor-blockade/hypoxia PH	Continuous CNP infusion	Negative CNP ligand-infusion evidence; not direct NPR-C/NPR3 manipulation	CNP did not attenuate pulmonary hypertension, right ventricular hypertrophy, or vascular remodeling in either model.	Important negative study showing phenotype- and model-dependence.
Human broader PH biomarker study	Kaiser et al. 2015 [15]	Prospective pilot clinical biomarker study	Patients with precapillary pulmonary hypertension and controls	NT-proCNP and other circulating natriuretic peptides	Ligand-level biomarker evidence in broader precapillary PH; not PH-CLD-specific and no NPR-C/NPR3 assay	NT-proCNP was associated mainly with right atrial pressure and renal function rather than robustly with pulmonary vascular haemodynamics.	Indirect human PH evidence; not specific to chronic lung disease or NPR-C/NPR3.
Human receptor-level hypothesis/genetic signal	Egom et al. 2019 [2]	Human report with mechanistic synthesis	Pulmonary arterial hypertension in relation to NPR-C/NPR3 pathway	NPR-C mutation/signaling pathway	Receptor-level NPR-C/NPR3 hypothesis/genetic-pathway evidence; indirect for PH-CLD	The study advanced NPR-C/NPR3 as a candidate receptor-level pathway in pulmonary arterial hypertension and vascular remodeling.	Human receptor-focused evidence, but indirect for Group 3 PH-CLD.
Acute lung injury and inflammation	Harrington et al. 2022 [18]	Transgenic mouse acute lung injury model	Npr3 wild-type, heterozygous, and knockout mice exposed to bacterial lung injury	Npr3/NPR-C gene dosage and ANP response	Direct NPR-C/Npr3 gene-dosage lung inflammation evidence	Functional NPR-C expression limited neutrophil recruitment and inflammatory cytokine responses during acute lung injury.	Receptor-specific lung inflammation evidence relevant to chronic lung disease mechanisms.
Acute lung injury and obesity	Guarino et al. 2023 [19]	Genetic mouse model of acute lung injury	Npr3 knockout and wild-type mice under diet-induced obesity conditions	Npr3 deletion	Direct Npr3 deletion lung injury evidence	Deletion of Npr3 increased severity of acute lung injury in obese mice.	Supports NPR-C as a modifier of lung injury susceptibility; indirect for PH-CLD.
Human pulmonary vascular cellular mechanism	Dabral et al. 2024 [24]	Human PAH pericyte mechanistic study	Pericytes from patients with pulmonary arterial hypertension	CNP/GC-B/cGMP/FoxO3 signaling	CNP/NPR-B/GC-B/cGMP pathway evidence; not NPR-C-specific	CNP signaling attenuated PAH pericyte proliferation, migration, and smooth muscle-like transdifferentiation.	Mechanistic support for CNP receptor biology in remodeling; primarily NPR-B/GC-B rather than NPR-C.
Experimental PH and NPR3 signaling	Sun et al. 2024 [20]	Preclinical pulmonary hypertension and cellular signaling study	Pulmonary hypertension models and pulmonary vascular cells	Musclin-NPR3/AKT/mTORC1 pathway	Direct NPR3-centered musclin/NPR3 signaling evidence	Musclin attenuated glycolysis, oxidative stress, and pulmonary hypertension through the NPR3/AKT/mTORC1 pathway.	Direct NPR3-centered PH mechanism; requires validation in PH-CLD cohorts.
Human clinical biomarker / PH-CLD	Avdeev et al. 2025 [9]	Clinical COPD pulmonary hypertension biomarker study	Patients with COPD with and without pulmonary hypertension	Plasma NT-proCNP	Ligand-only NT-proCNP biomarker evidence in COPD-PH; no NPR-C/NPR3 receptor assay	NT-proCNP was higher in COPD-associated pulmonary hypertension and showed diagnostic discrimination in a pilot cohort.	Most direct PH-CLD biomarker evidence; no direct NPR-C/NPR3 receptor assay.
Human pulmonary vascular cellular metabolism	Noh et al. 2025 [25]	Human PAH pericyte metabolic study	Pericytes from patients with pulmonary arterial hypertension	CNP/cGMP signaling, glycolysis, de novo pyrimidine synthesis	CNP/GC-B/cGMP metabolic pathway evidence; not NPR-C-specific	CNP attenuated PDGF-driven glycolysis and de novo pyrimidine synthesis in PAH pericytes.	Supports anti-proliferative CNP biology in vascular remodeling; receptor specificity beyond GC-B/cGMP remains unresolved.
Experimental PH and right ventricular remodeling	Dignam et al. 2025 [26]	Genetic and pharmacologic mouse pulmonary hypertension study	Endothelial CNP-deficient, cardiomyocyte CNP-deficient, and NPR-C-deficient mice exposed to SuHx	Endogenous CNP, NPR-B/NPR-C signaling, exogenous CNP	Direct preclinical CNP/NPR-C evidence, including NPR-C deletion and RV remodeling	Endothelial CNP protected against PH; global NPR-C deletion worsened right ventricular hypertrophy and fibrosis; exogenous CNP reduced RVSP.	Strong preclinical CNP/NPR-C evidence for pulmonary vascular and RV remodeling.
Experimental CNP receptor pathway	Yanagisawa et al. 2026 [27]	Experimental pulmonary arterial hypertension study	Mouse pulmonary hypertension models with vascular CNP/GC-B pathway manipulation	Endothelial CNP/guanylyl cyclase-B signaling with NPR-C pathway context	Primarily endothelial CNP/GC-B pathway evidence; NPR-C is contextual rather than central	Endothelial CNP/GC-B signaling was implicated in protection against pulmonary arterial hypertension progression.	Supports receptor-specific CNP-pathway relevance; NPR-B/GC-B is central, with NPR-C as pathway context.

Abbreviations: CNP, C-type natriuretic peptide; COPD, chronic obstructive pulmonary disease; FGF, fibroblast growth factor; GC-B, guanylyl cyclase-B; ILD, interstitial lung disease; MCT, monocrotaline; NPR-C, natriuretic peptide receptor-C; NPR3, natriuretic peptide receptor 3 gene; PAH, pulmonary arterial hypertension; PDGF, platelet-derived growth factor; PH, pulmonary hypertension; PH-CLD, pulmonary hypertension associated with chronic lung disease; RV, right ventricle; RVH, right ventricular hypertrophy; RVSP, right ventricular systolic pressure; SuHx, Sugen-hypoxia; VEGF, vascular endothelial growth factor.

**Table 2:** Distribution of included original studies by primary evidence domain.

Primary Evidence Domain	Number of Studies	Representative Studies	Directness for PH-CLD Interpretation
Human clinical biomarker/phenotyping	3	Cargill [14], Kaiser [15], Avdeev [9]	Highest directness when COPD-PH/cor pulmonale is present; limited receptor specificity.
Human receptor-level/genetic signal	1	Egom [2]	Human receptor-centered evidence, but indirect for Group 3 PH.
Hypoxia and pulmonary vascular receptor regulation	3	Klinger [17], Sun 2000 [20], Sun 2001 [21]	Mechanistically relevant to Group 3 PH; preclinical/cellular.
Experimental PH/RV remodeling	4	Itoh [22], Casserly [8], Dignam [26], Yanagisawa [27]	High mechanistic relevance; model-dependent clinical translation.
Chronic lung injury/fibrosis	3	Murakami [23], Harrington [18], Guarino [19]	Relevant to ILD/fibrotic lung disease mechanisms; PH not always directly measured.
Pulmonary vascular cellular/metabolic mechanisms	3	Dabral [24], Noh [25], Sun 2024 [20]	Strong mechanistic resolution; limited direct clinical applicability.

**Table 3:** Evidence-to-translation matrix for NPR-C/NPR3 in pulmonary hypertension associated with chronic lung disease.

Translational Domain	What the Included Evidence Supports	Current Limitation	Minimum Next Step
Diagnosis	Clinical biomarker studies suggest CNP/NT-proCNP signals may reflect pulmonary vascular stress in COPD-PH and broader precapillary PH.	Direct NPR-C/NPR3 receptor validation in PH-CLD is absent; echocardiographic PH definitions predominate in the COPD biomarker study.	Prospective PH-CLD cohorts with right heart catheterization, CNP/NT-proCNP, BNP/NT-proBNP, NPR3 expression/genotype, CT, echocardiography, and exercise testing.
Prognosis	Preclinical studies link CNP/NPR-C biology to pulmonary vascular remodeling and RV hypertrophy/fibrosis; human prognostic data are limited.	No prospective PH-CLD study links NPR3/NPR-C measures to mortality, hospitalization, clinical worsening, transplant referral, or RV failure.	Longitudinal outcome studies with standardized PH-CLD endpoints and multivariable models testing incremental prognostic value.
Mechanistic phenotyping	Hypoxia and growth-factor signaling can alter NPR-C expression; CNP receptor signaling modifies smooth muscle, endothelial, fibroblast, inflammatory, and pericyte behavior.	CNP effects are often not receptor-specific; many studies emphasize NPR-B/GC-B rather than NPR-C/NPR3.	Cell-specific receptor mapping in COPD-PH and ILD-PH tissue, with loss/gain-of-function experiments and receptor-selective pharmacology.
Therapeutic targeting	Some experimental models show CNP-related protection; others show no effect, indicating disease-model dependence.	No human intervention study has tested NPR-C/NPR3 modulation in PH-CLD.	Preclinical target-validation studies followed by safety-focused early-phase trials only after receptor specificity and phenotype selection are established.

**Table 4:** Evidence gaps and proposed approaches for future NPR-C/NPR3 research in PH-CLD.

Gap	Why it Matters	Proposed Approach
Human PH-CLD evidence	Only sparse clinical data exist in COPD-PH or cor pulmonale, and most work measures ligand fragments rather than receptor biology.	Create multicenter prospective COPD-PH and ILD-PH cohorts with invasive haemodynamics when clinically indicated.
Receptor specificity	CNP can signal through NPR-B/GC-B and NPR-C; several studies do not distinguish these pathways.	Use receptor-selective assays, NPR3/NPR2 loss-of-function models, cell-specific expression maps, and receptor activity readouts.
Right ventricular translation	Preclinical evidence implicates CNP/NPR-C biology in RV hypertrophy and fibrosis, but human RV data are minimal.	Pair biomarkers and receptor biology with cardiac MRI, echocardiographic RV-PA coupling, RV strain, and longitudinal outcomes.
Chronic lung disease phenotyping	COPD, ILD, CPFE, sleep-disordered breathing, and hypoventilation likely have different vascular mechanisms.	Pre-specify PH-CLD subtypes and use PFT, DLCO, ABG, CT parenchymal/vascular markers, and oxygenation metrics.
Therapeutic readiness	Protective effects differ across experimental models and cannot yet justify clinical targeting.	Establish dose, timing, route, receptor mechanism, and safety before human trials of NPR-C/NPR3 modulation.

**Table 5:** Proposed minimum dataset for a prospective PH-CLD NPR-C/NPR3 translational cohort.

Data Domain	Recommended Elements
Clinical phenotype	COPD, ILD, CPFE, sleep-disordered breathing/hypoventilation, chronic hypoxic lung disease; smoking history; comorbid left-heart disease; renal function
Pulmonary vascular phenotype	Right heart catheterization when clinically indicated; mPAP, PVR, PAWP, RAP, cardiac output/index; echocardiographic probability; RVSP; RV-PA coupling
Lung disease severity	Spirometry, lung volumes, DLCO, arterial blood gases, oxygen requirement, CT emphysema/fibrosis extent, pulmonary artery/aorta ratio
Natriuretic peptide axis	BNP/NT-proBNP, CNP/NT-proCNP, NPR3 genotype, NPR3 expression if tissue/blood RNA available, NPR-C protein or receptor-function assay where feasible
Right ventricular outcomes	Cardiac MRI or echocardiographic RV size/function, RV strain, RV hypertrophy, RV fibrosis markers, exercise capacity
Longitudinal endpoints	Clinical worsening, hospitalization, mortality, transplant referral, oxygen escalation, pulmonary rehabilitation response, treatment response

## Data Availability

No new data were created or analyzed in this study. Data sharing is not applicable to this article. The search strategy and evidence map are provided in the manuscript and Supplementary Materials.

## Supplementary Materials

The following supporting information can be downloaded at: https://www.mdpi.com/article/10.3390/jcm15197429/s1, Supplementary File S1: Completed PRISMA-ScR Checklist; Supplementary File S2: Electronic Search Strategy.
