# Supplementary material for: Natriuretic Peptide Receptor-C (NPR3) in Pulmonary Hypertension Associated with Chronic Lung Disease: A PRISMA-ScR Scoping Review of Diagnostic, Prognostic, Mechanistic, and Therapeutic Evidence
Source: J Clin Med. 2026 Sep 24;15(19):7429. doi: 10.3390/jcm15197429 (PMC13666394; doi:10.3390/jcm15197429)
Supplement: Supplementary file 1 [file jcm-15-07429-s001.zip › Supplementary File S1 – Completed PRISMA-ScR Checklist.pdf]

Supplementary File S1. Completed PRISMA-ScR Checklist

Natriuretic Peptide Receptor-C (NPR3) in Pulmonary Hypertension Associated with Chronic Lung Disease: A PRISMA-ScR Scoping Review of Diagnostic, Prognostic, Mechanistic, and Therapeutic Evidence

**Manuscript ID**	jcm-4360364
**Journal**	Journal of Clinical Medicine
**Special Issue**	Pulmonary Hypertension and Chronic Lung Disease: Diagnosis and Management
**Reporting guideline**	PRISMA Extension for Scoping Reviews (PRISMA-ScR)
**Checklist date**	21 May 2026

Compliance statement. This completed checklist maps the PRISMA-ScR reporting items to the revised manuscript. The manuscript reports compliance with PRISMA-ScR in the Methods section, includes the study-selection process as Figure 1, and provides the complete electronic search strategy in Supplementary File S2.

Completed PRISMA-ScR checklist

Section	No.	Checklist item	PRISMA-ScR requirement	Status	Manuscript location and response
Title	1	Title	Identify the report as a scoping review.	Reported	Title. The title explicitly identifies the manuscript as a PRISMA-ScR scoping review.
Abstract	2	Structured summary	Provide a structured summary that includes the rationale, objectives, eligibility criteria, sources of evidence, charting methods, results, and conclusions relevant to a scoping review.	Reported	Abstract. The structured abstract includes Background/Objectives, Methods, Results, and Conclusions, including PRISMA counts and the number of included original studies.
Introduction	3	Rationale	Describe the rationale for the review in the context of what is already known and explain why the review questions or objectives are suited to a scoping review approach.	Reported	Introduction. The rationale describes the clinical and translational gap in NPR-C/NPR3 biology within pulmonary hypertension associated with chronic lung disease.
Introduction	4	Objectives	Provide an explicit statement of the questions and objectives being addressed with reference to key elements such as population, concepts, and context.	Reported	Introduction, final paragraph; Methods 2.1. The manuscript states that the review maps original empirical evidence on NPR-C/NPR3 and related C-type natriuretic peptide biology in pulmonary hypertension associated with chronic lung disease.

Section	No.	Checklist item	PRISMA-ScR requirement	Status	Manuscript location and response
Methods	5	Protocol and registration	Indicate whether a review protocol exists; state where it can be accessed; and provide registration information if available.	Reported	Methods 2.1. A protocol was developed before screening. The protocol was not registered; eligibility criteria, search strategies, screening procedures, and data-charting procedures were defined before evidence selection.
Methods	6	Eligibility criteria	Specify characteristics of sources of evidence used as eligibility criteria and provide a rationale.	Reported	Methods 2.2. Eligibility criteria are defined using the Population-Concept-Context framework and restrict inclusion to original empirical studies relevant to NPR-C/NPR3, pulmonary hypertension, chronic lung disease, pulmonary vascular remodeling, or right ventricular remodeling.
Methods	7	Information sources	Describe all information sources searched or consulted and the date of the last search.	Reported	Methods 2.3; Appendix A. PubMed/MEDLINE and APA PsycINFO were searched from database inception to 18 May 2026. A Web of Science Core Collection strategy was developed for reproducibility but was not executed and was not included in the final PRISMA counts.
Methods	8	Search	Present the full electronic search strategy for at least one database, including any limits used, such that the search could be repeated.	Reported	Appendix A. Complete PubMed/MEDLINE and APA PsycINFO search strategies are provided, including Boolean syntax, field tags, coverage, limits, and database-specific syntax conventions.
Methods	9	Selection of sources of evidence	State the process for selecting sources of evidence, including screening and eligibility assessment.	Reported	Methods 2.5. Titles and abstracts were screened first, followed by full-text eligibility assessment using the same Population-Concept-Context framework.
Methods	10	Data charting process	Describe the methods of charting data from included sources and any processes used to obtain and confirm data.	Reported	Methods 2.6. Data were charted using a standardized extraction form developed before full-text assessment, piloted on included studies, and verified by a second reviewer.
Methods	11	Data items	List and define all variables for which data were sought and any assumptions or simplifications made.	Reported	Methods 2.6. The manuscript lists bibliographic, clinical, model, exposure, outcome, mechanistic, right ventricular, management-relevance, and limitation variables extracted from included studies.
Methods	12	Critical appraisal of individual sources of evidence	If done, provide the rationale and methods for critical appraisal of included sources of evidence.	Addressed; formal risk-of-bias appraisal not performed	Methods 2.8; Discussion 4.6. Formal risk-of-bias appraisal was not used as an inclusion criterion because the review was designed to map the evidence landscape. Methodological features relevant to interpretation were charted and discussed.
Methods	13	Synthesis of results	Describe the methods of handling and summarizing charted data.	Reported	Methods 2.7. A descriptive and narrative synthesis was performed. Evidence was summarized numerically and thematically by diagnostic relevance, prognostic relevance, mechanisms, therapeutic or management relevance, and translational gaps.
Results	14	Selection of sources of evidence	Give numbers of sources screened, assessed for eligibility, and included, with reasons for exclusions at each stage, ideally using a flow diagram.	Reported	Results 3.1; Figure 1. The manuscript reports 72 records identified, 2 duplicates removed, 70 records screened, 28 reports assessed for eligibility, 11 full-text exclusions with reasons, and 17 included original studies.

Section	No.	Checklist item	PRISMA-ScR requirement	Status	Manuscript location and response
Results	15	Characteristics of sources of evidence	For each source of evidence, present characteristics for which data were charted and provide citations.	Reported	Results 3.2; Table 1. The evidence map reports study domain, design, population or model, NPR-C/NPR3 or CNP construct, key findings, and interpretation for PH-CLD.
Results	16	Critical appraisal within sources of evidence	If done, present data on critical appraisal of included sources of evidence.	Not applicable; formal critical appraisal not conducted	Methods 2.8; Discussion 4.6. No formal risk-of-bias appraisal was performed. Study design, phenotyping quality, receptor-specific measurement, and other interpretive limitations were charted and incorporated into the synthesis.
Results	17	Results of individual sources of evidence	For each included source, present relevant data that were charted and that relate to the review questions and objectives.	Reported	Results 3.3-3.7; Table 1. The manuscript summarizes findings from human biomarker studies, receptor-expression studies, experimental pulmonary hypertension models, chronic lung injury and fibrosis models, and pulmonary vascular cellular studies.
Results	18	Synthesis of results	Summarize or present charting results as they relate to the review questions and objectives.	Reported	Results 3.8; Tables 2-5; Figures 2-3. The synthesis summarizes evidence domains, translational implications, evidence gaps, and a conceptual framework for NPR-C/NPR3 in PH-CLD.
Discussion	19	Summary of evidence	Summarize the main results, including an overview of concepts, themes, and types of evidence, and link findings to the review questions and objectives.	Reported	Discussion 4.1-4.5. The discussion summarizes principal findings, interpretation in relation to prior syntheses, gaps, conceptual framework, and research agenda.
Discussion	20	Limitations	Discuss limitations of the scoping review process.	Reported	Discussion 4.6. The limitations section addresses database scope, indirect evidence, receptor-specific uncertainty, absence of formal risk-of-bias appraisal, and heterogeneity precluding quantitative synthesis.
Discussion	21	Conclusions	Provide a general interpretation of the results with respect to the review questions and objectives, as well as potential implications or next steps.	Reported	Section 5. The conclusion states that NPR-C/NPR3 is biologically plausible but not clinically validated in PH-CLD and identifies prospective human validation as the main research priority.
Funding	22	Funding	Describe sources of funding for the scoping review and, where applicable, the role of funders.	Reported	Back matter funding statement. The scoping review received no external funding. Funding sources of included studies were not used as eligibility criteria or as a synthesis variable.

Checklist reference. Tricco AC, Lillie E, Zarin W, O'Brien KK, Colquhoun H, Levac D, Moher D, Peters MDJ, Horsley T, Weeks L, et al. PRISMA Extension for Scoping Reviews (PRISMA-ScR): Checklist and Explanation. Annals of Internal Medicine. 2018;169:467-473.
