# Supplementary material for: Natriuretic Peptide Receptor-C (NPR3) in Pulmonary Hypertension Associated with Chronic Lung Disease: A PRISMA-ScR Scoping Review of Diagnostic, Prognostic, Mechanistic, and Therapeutic Evidence
Source: J Clin Med. 2026 Sep 24;15(19):7429. doi: 10.3390/jcm15197429 (PMC13666394; doi:10.3390/jcm15197429)
Supplement: Supplementary file 1 [file jcm-15-07429-s001.zip › Supplementary File S2 – Electronic Search Strategy.docx.pdf]

Supplementary File S2. Electronic Search Strategy

Natriuretic Peptide Receptor-C/NPR3 in Pulmonary Hypertension Associated with Chronic Lung Disease

S.1. Reporting statement and search scope

This appendix reports the complete electronic search strategies for PubMed/MEDLINE and APA PsycINFO via APA PsycNet, which were used for the final PRISMA counts. A Web of Science Core Collection strategy is also provided for reproducibility, but that database search was not executed and was not included in the final PRISMA counts. The strategy was prepared to support PRISMA-S search reporting and PRISMA-ScR manuscript reporting and was designed to retrieve evidence on natriuretic peptide receptor-C/NPR3 biology in pulmonary hypertension associated with chronic lung disease, while retaining broader pulmonary vascular and right ventricular mechanistic evidence for screening.

Coverage: database inception to present, defined for this appendix as 18 May 2026.

Limits and filters: no limits or filters by date, language, publication type, age group, or species were applied at the electronic search stage.

Reviews at search stage: reviews, editorials, and other non-original records were not excluded during database searching. They may be used for citation tracking, but only original empirical studies are eligible for inclusion in the evidence synthesis according to the protocol.

Search structure: the primary search combined three concepts: NPR-C/NPR3 biology, pulmonary hypertension or pulmonary vascular/right ventricular disease, and chronic lung disease or hypoxia-related lung disease. A supplementary sensitivity search combined NPR-C/NPR3 biology with pulmonary hypertension or pulmonary vascular/right ventricular disease without the chronic lung disease block.

S.2. Databases, platforms, coverage, and search fields

Database	Platform/interface	Coverage	Search field(s)	Limits
PubMed/MEDLINE	NCBI PubMed	Database inception to 18 May 2026	MeSH and title/abstract terms: [Mesh], [tiab]	None
APA PsycINFO	APA PsycNet Advanced Search	Database inception to 18 May 2026	Any Field search using Boolean operators, phrases, parentheses, and truncation	None
Web of Science Core Collection	Clarivate Web of Science Advanced Search; strategy developed for reproducibility	Not executed for final PRISMA counts	Topic field: TS=	Not applicable to final counts

S.3. Field tags and syntax conventions

Element	Meaning in this appendix	Rationale
[Mesh]	PubMed/MEDLINE Medical Subject Headings.	Used to retrieve indexed biomedical concepts, including pulmonary hypertension, chronic lung disease, hypoxia, and natriuretic peptide receptor-related headings.
[tiab]	PubMed title/abstract field.	Used to capture newer records not yet indexed with MeSH and records using precise molecular terminology such as NPR3, NPR-C, ANPRC, or natriuretic peptide clearance receptor.
Any Field	APA PsycINFO via APA PsycNet broad search field.	Used because the topic may appear in titles, abstracts, keywords, or indexed fields and because controlled vocabulary may not capture receptor-level cardiopulmonary terminology consistently.
TS=	Web of Science Topic field.	Searches title, abstract, author keywords, and Keywords Plus fields in Web of Science Core Collection.

Quotation marks	Exact phrase searching.	Used for multiword expressions such as "pulmonary hypertension" and "natriuretic peptide receptor C".
OR	Combines synonyms within a concept block.	Broadens retrieval by capturing alternative spellings, abbreviations, and related terms.
AND	Combines different concept blocks.	Narrows retrieval to records that overlap NPR-C/NPR3 biology with pulmonary vascular disease and, in the primary search, chronic lung disease.
*	Truncation where supported by the database.	Captures variant word endings, such as peptide and peptides.
NOT	Not used.	Avoided to reduce the risk of unintentionally excluding relevant records.

S.4. Boolean logic

Primary search: (*NPR-C/NPR3 and natriuretic peptide receptor biology*) AND (*pulmonary hypertension, pulmonary vascular remodeling, or right ventricular disease*) AND (*chronic lung disease, hypoxia, fibrosis, emphysema, COPD, ILD, sleep-disordered breathing, or experimental lung disease models*).

Supplementary sensitivity search: (*NPR-C/NPR3 and natriuretic peptide receptor biology*) AND (*pulmonary hypertension, pulmonary vascular remodeling, or right ventricular disease*).

S.5. PubMed/MEDLINE search strategy

Database: PubMed/MEDLINE via NCBI PubMed.

Coverage: database inception to 18 May 2026.

Fields: MeSH terms and title/abstract terms using [Mesh] and [tiab].

Limits: none.

S.5.1. Primary PubMed/MEDLINE search

```
(
  "Receptors, Atrial Natriuretic Factor"[Mesh]
  OR "Natriuretic Peptide, C-Type"[Mesh]
  OR "natriuretic peptide receptor C"[tiab]
  OR "natriuretic peptide receptor-C"[tiab]
  OR "natriuretic peptide receptor 3"[tiab]
  OR "natriuretic peptide receptor-3"[tiab]
  OR "natriuretic peptide receptor type C"[tiab]
  OR "atrial natriuretic peptide receptor C"[tiab]
  OR "atrial natriuretic peptide receptor-C"[tiab]
  OR "atrial natriuretic peptide receptor 3"[tiab]
  OR "atrial natriuretic peptide receptor type C"[tiab]
  OR "atrionatriuretic peptide receptor C"[tiab]
  OR "ANP receptor C"[tiab]
  OR "ANP receptor-C"[tiab]
  OR "ANF receptor C"[tiab]
  OR "NP receptor C"[tiab]
  OR "NP receptor-C"[tiab]
  OR "natriuretic peptide clearance receptor"[tiab]
  OR "atrial natriuretic peptide clearance receptor"[tiab]
  OR "NPR-C"[tiab]
  OR "NPR C"[tiab]
  OR "NPR-3"[tiab]
  OR NPRC[tiab]
  OR NPR3[tiab]
  OR ANPRC[tiab]
  OR "ANPR-C"[tiab]
  OR "ANP-C"[tiab]
  OR (
    ("clearance receptor"[tiab] OR "clearance receptors"[tiab])
    AND (natriuretic[tiab] OR "atrial natriuretic"[tiab] OR "C-type natriuretic"[tiab])
  )
  OR "C-type natriuretic peptide"[tiab]
  OR "C type natriuretic peptide"[tiab]
  OR NT-proCNP[tiab]
```

```
  OR "NT proCNP"[tiab]
  OR "amino-terminal pro-C-type natriuretic peptide"[tiab]
  OR (
    CNP[tiab]
    AND (natriuretic[tiab] OR peptide*[tiab] OR receptor*[tiab])
  )
)
AND
(
  "Hypertension, Pulmonary"[Mesh]
  OR "Pulmonary Arterial Hypertension"[Mesh]
  OR "Pulmonary Circulation"[Mesh]
  OR "Pulmonary Heart Disease"[Mesh]
  OR "Vascular Remodeling"[Mesh]
  OR "Ventricular Dysfunction, Right"[Mesh]
  OR "pulmonary hypertension"[tiab]
  OR "pulmonary arterial hypertension"[tiab]
  OR "group 3 pulmonary hypertension"[tiab]
  OR "group III pulmonary hypertension"[tiab]
  OR "pulmonary vascular disease"[tiab]
  OR "pulmonary vascular remodeling"[tiab]
  OR "pulmonary vascular remodelling"[tiab]
  OR "pulmonary artery remodeling"[tiab]
  OR "pulmonary artery remodelling"[tiab]
  OR "pulmonary arterial remodeling"[tiab]
  OR "pulmonary arterial remodelling"[tiab]
  OR "pulmonary vascular resistance"[tiab]
  OR "mean pulmonary arterial pressure"[tiab]
  OR mPAP[tiab]
  OR "right heart catheterization"[tiab]
  OR "right heart catheterisation"[tiab]
  OR "right ventricular"[tiab]
  OR "right ventricle"[tiab]
  OR "right ventricular dysfunction"[tiab]
  OR "right ventricular hypertrophy"[tiab]
  OR "right ventricular remodeling"[tiab]
  OR "right ventricular remodelling"[tiab]
  OR "cor pulmonale"[tiab]
  OR "pulmonary artery smooth muscle"[tiab]
  OR "pulmonary arterial smooth muscle"[tiab]
  OR PASMC*[tiab]
  OR "pulmonary endothelial"[tiab]
  OR "pulmonary artery endothelial"[tiab]
  OR "pulmonary arterial endothelial"[tiab]
  OR PAEC[tiab]
  OR monocrotaline[tiab]
  OR SU5416[tiab]
  OR Sugen[tiab]
  OR "Sugen-hypoxia"[tiab]
)
AND
(
  "Lung Diseases"[Mesh]
  OR "Lung Diseases, Obstructive"[Mesh]
  OR "Pulmonary Disease, Chronic Obstructive"[Mesh]
  OR "Pulmonary Emphysema"[Mesh]
  OR "Lung Diseases, Interstitial"[Mesh]
  OR "Pulmonary Fibrosis"[Mesh]
  OR "Idiopathic Pulmonary Fibrosis"[Mesh]
  OR "Hypoxia"[Mesh]
  OR "Sleep Apnea Syndromes"[Mesh]
  OR "Bronchopulmonary Dysplasia"[Mesh]
  OR "Respiratory Insufficiency"[Mesh]
  OR "chronic lung disease"[tiab]
  OR "chronic respiratory disease"[tiab]
  OR COPD[tiab]
  OR "chronic obstructive pulmonary disease"[tiab]
  OR emphysema[tiab]
  OR "interstitial lung disease"[tiab]
  OR ILD[tiab]
  OR "pulmonary fibrosis"[tiab]
  OR "idiopathic pulmonary fibrosis"[tiab]
  OR IPF[tiab]
  OR "combined pulmonary fibrosis and emphysema"[tiab]
  OR CPFE[tiab]
```

```
  OR hypoxia[tiab]
  OR hypoxic[tiab]
  OR hypoxemia[tiab]
  OR hypoxaemia[tiab]
  OR "chronic hypoxia"[tiab]
  OR "hypoxia-induced"[tiab]
  OR "lung injury"[tiab]
  OR bleomycin[tiab]
  OR "cigarette smoke"[tiab]
  OR "smoke exposure"[tiab]
  OR "sleep apnea"[tiab]
  OR "sleep apnoea"[tiab]
  OR "sleep-disordered breathing"[tiab]
  OR "sleep disordered breathing"[tiab]
  OR hypoventilation[tiab]
  OR "obesity hypoventilation"[tiab]
  OR "bronchopulmonary dysplasia"[tiab]
)
```

S.5.2. Supplementary PubMed/MEDLINE sensitivity search

```
(
  "Receptors, Atrial Natriuretic Factor"[Mesh]
  OR "Natriuretic Peptide, C-Type"[Mesh]
  OR "natriuretic peptide receptor C"[tiab]
  OR "natriuretic peptide receptor-C"[tiab]
  OR "natriuretic peptide receptor 3"[tiab]
  OR "natriuretic peptide receptor-3"[tiab]
  OR "natriuretic peptide receptor type C"[tiab]
  OR "atrial natriuretic peptide receptor C"[tiab]
  OR "atrial natriuretic peptide receptor-C"[tiab]
  OR "atrial natriuretic peptide receptor 3"[tiab]
  OR "atrial natriuretic peptide receptor type C"[tiab]
  OR "atrionatriuretic peptide receptor C"[tiab]
  OR "ANP receptor C"[tiab]
  OR "ANP receptor-C"[tiab]
  OR "ANF receptor C"[tiab]
  OR "NP receptor C"[tiab]
  OR "NP receptor-C"[tiab]
  OR "natriuretic peptide clearance receptor"[tiab]
  OR "atrial natriuretic peptide clearance receptor"[tiab]
  OR "NPR-C"[tiab]
  OR "NPR C"[tiab]
  OR "NPR-3"[tiab]
  OR NPRC[tiab]
  OR NPR3[tiab]
  OR ANPRC[tiab]
  OR "ANPR-C"[tiab]
  OR "ANP-C"[tiab]
  OR (
    ("clearance receptor"[tiab] OR "clearance receptors"[tiab])
    AND (natriuretic[tiab] OR "atrial natriuretic"[tiab] OR "C-type natriuretic"[tiab])
  )
  OR "C-type natriuretic peptide"[tiab]
  OR "C type natriuretic peptide"[tiab]
  OR NT-proCNP[tiab]
  OR "NT proCNP"[tiab]
  OR "amino-terminal pro-C-type natriuretic peptide"[tiab]
  OR (
    CNP[tiab]
    AND (natriuretic[tiab] OR peptide*[tiab] OR receptor*[tiab])
  )
)
AND
(
  "Hypertension, Pulmonary"[Mesh]
  OR "Pulmonary Arterial Hypertension"[Mesh]
  OR "Pulmonary Circulation"[Mesh]
  OR "Pulmonary Heart Disease"[Mesh]
  OR "Vascular Remodeling"[Mesh]
  OR "Ventricular Dysfunction, Right"[Mesh]
  OR "pulmonary hypertension"[tiab]
  OR "pulmonary arterial hypertension"[tiab]
  OR "pulmonary vascular disease"[tiab]
  OR "pulmonary vascular remodeling"[tiab]
```

```
  OR "pulmonary vascular remodelling"[tiab]
  OR "pulmonary artery remodeling"[tiab]
  OR "pulmonary artery remodelling"[tiab]
  OR "pulmonary arterial remodeling"[tiab]
  OR "pulmonary arterial remodelling"[tiab]
  OR "pulmonary vascular resistance"[tiab]
  OR "mean pulmonary arterial pressure"[tiab]
  OR mPAP[tiab]
  OR "right heart catheterization"[tiab]
  OR "right heart catheterisation"[tiab]
  OR "right ventricular"[tiab]
  OR "right ventricle"[tiab]
  OR "right ventricular dysfunction"[tiab]
  OR "right ventricular hypertrophy"[tiab]
  OR "right ventricular remodeling"[tiab]
  OR "right ventricular remodelling"[tiab]
  OR "cor pulmonale"[tiab]
  OR "pulmonary artery smooth muscle"[tiab]
  OR "pulmonary arterial smooth muscle"[tiab]
  OR PASMC*[tiab]
  OR "pulmonary endothelial"[tiab]
  OR "pulmonary artery endothelial"[tiab]
  OR "pulmonary arterial endothelial"[tiab]
  OR PAEC[tiab]
  OR monocrotaline[tiab]
  OR SU5416[tiab]
  OR Sugen[tiab]
  OR "Sugen-hypoxia"[tiab]
)
```

S.6. APA PsycINFO search strategy

Database: APA PsycINFO via APA PsycNet.

Coverage: database inception to 18 May 2026.

Fields: Advanced Search, Any Field.

Limits: none.

S.6.1. Primary APA PsycINFO search

```
(
  "natriuretic peptide receptor C"
  OR "natriuretic peptide receptor-C"
  OR "natriuretic peptide receptor 3"
  OR "natriuretic peptide receptor-3"
  OR "natriuretic peptide receptor type C"
  OR "atrial natriuretic peptide receptor C"
  OR "atrial natriuretic peptide receptor-C"
  OR "atrial natriuretic peptide receptor 3"
  OR "atrial natriuretic peptide receptor type C"
  OR "atrionatriuretic peptide receptor C"
  OR "ANP receptor C"
  OR "ANP receptor-C"
  OR "ANF receptor C"
  OR "NP receptor C"
  OR "NP receptor-C"
  OR "natriuretic peptide clearance receptor"
  OR "atrial natriuretic peptide clearance receptor"
  OR "NPR-C"
  OR "NPR C"
  OR "NPR-3"
  OR NPRC
  OR NPR3
  OR ANPRC
  OR "ANPR-C"
  OR "ANP-C"
  OR (("clearance receptor" OR "clearance receptors") AND (natriuretic OR "atrial natriuretic" OR "C-
type natriuretic"))
  OR "C-type natriuretic peptide"
  OR "C type natriuretic peptide"
  OR NT-proCNP
  OR "NT proCNP"
  OR "amino-terminal pro-C-type natriuretic peptide"
```

```
  OR (CNP AND (natriuretic OR peptide* OR receptor*))
)
AND
(
  "pulmonary hypertension"
  OR "pulmonary arterial hypertension"
  OR "group 3 pulmonary hypertension"
  OR "group III pulmonary hypertension"
  OR "pulmonary vascular disease"
  OR "pulmonary vascular remodeling"
  OR "pulmonary vascular remodelling"
  OR "pulmonary artery remodeling"
  OR "pulmonary artery remodelling"
  OR "pulmonary arterial remodeling"
  OR "pulmonary arterial remodelling"
  OR "pulmonary vascular resistance"
  OR "mean pulmonary arterial pressure"
  OR mPAP
  OR "right heart catheterization"
  OR "right heart catheterisation"
  OR "right ventricular"
  OR "right ventricle"
  OR "right ventricular dysfunction"
  OR "right ventricular hypertrophy"
  OR "right ventricular remodeling"
  OR "right ventricular remodelling"
  OR "cor pulmonale"
  OR "pulmonary artery smooth muscle"
  OR "pulmonary arterial smooth muscle"
  OR PASMC*
  OR "pulmonary endothelial"
  OR "pulmonary artery endothelial"
  OR "pulmonary arterial endothelial"
  OR PAEC
  OR monocrotaline
  OR SU5416
  OR Sugen
  OR "Sugen-hypoxia"
)
AND
(
  "chronic lung disease"
  OR "chronic respiratory disease"
  OR COPD
  OR "chronic obstructive pulmonary disease"
  OR emphysema
  OR "interstitial lung disease"
  OR ILD
  OR "pulmonary fibrosis"
  OR "idiopathic pulmonary fibrosis"
  OR IPF
  OR "combined pulmonary fibrosis and emphysema"
  OR CPFE
  OR hypoxia
  OR hypoxic
  OR hypoxemia
  OR hypoxaemia
  OR "chronic hypoxia"
  OR "hypoxia-induced"
  OR "lung injury"
  OR bleomycin
  OR "cigarette smoke"
  OR "smoke exposure"
  OR "sleep apnea"
  OR "sleep apnoea"
  OR "sleep-disordered breathing"
  OR "sleep disordered breathing"
  OR hypoventilation
  OR "obesity hypoventilation"
  OR "bronchopulmonary dysplasia"
)
```

S.6.2. Supplementary APA PsycINFO sensitivity search

```
(
```

```
  "natriuretic peptide receptor C"
  OR "natriuretic peptide receptor-C"
  OR "natriuretic peptide receptor 3"
  OR "natriuretic peptide receptor-3"
  OR "natriuretic peptide receptor type C"
  OR "atrial natriuretic peptide receptor C"
  OR "atrial natriuretic peptide receptor-C"
  OR "atrial natriuretic peptide receptor 3"
  OR "atrial natriuretic peptide receptor type C"
  OR "atrionatriuretic peptide receptor C"
  OR "ANP receptor C"
  OR "ANP receptor-C"
  OR "ANF receptor C"
  OR "NP receptor C"
  OR "NP receptor-C"
  OR "natriuretic peptide clearance receptor"
  OR "atrial natriuretic peptide clearance receptor"
  OR "NPR-C"
  OR "NPR C"
  OR "NPR-3"
  OR NPRC
  OR NPR3
  OR ANPRC
  OR "ANPR-C"
  OR "ANP-C"
  OR (("clearance receptor" OR "clearance receptors") AND (natriuretic OR "atrial natriuretic" OR "C-
type natriuretic"))
  OR "C-type natriuretic peptide"
  OR "C type natriuretic peptide"
  OR NT-proCNP
  OR "NT proCNP"
  OR "amino-terminal pro-C-type natriuretic peptide"
  OR (CNP AND (natriuretic OR peptide* OR receptor*))
)
AND
(
  "pulmonary hypertension"
  OR "pulmonary arterial hypertension"
  OR "pulmonary vascular disease"
  OR "pulmonary vascular remodeling"
  OR "pulmonary vascular remodelling"
  OR "pulmonary artery remodeling"
  OR "pulmonary artery remodelling"
  OR "pulmonary arterial remodeling"
  OR "pulmonary arterial remodelling"
  OR "pulmonary vascular resistance"
  OR "mean pulmonary arterial pressure"
  OR mPAP
  OR "right heart catheterization"
  OR "right heart catheterisation"
  OR "right ventricular"
  OR "right ventricle"
  OR "right ventricular dysfunction"
  OR "right ventricular hypertrophy"
  OR "right ventricular remodeling"
  OR "right ventricular remodelling"
  OR "cor pulmonale"
  OR "pulmonary artery smooth muscle"
  OR "pulmonary arterial smooth muscle"
  OR PASMC*
  OR "pulmonary endothelial"
  OR "pulmonary artery endothelial"
  OR "pulmonary arterial endothelial"
  OR PAEC
  OR monocrotaline
  OR SU5416
  OR Sugen
  OR "Sugen-hypoxia"
)
```

S.7. Developed Web of Science Core Collection search strategy, not included in final PRISMA counts

Database: Web of Science Core Collection via Clarivate Web of Science. This strategy was developed for reproducibility but was not executed and was not included in the final PRISMA counts.

Coverage: all years available through institutional subscription to 18 May 2026.

Fields: Topic search using TS=, which searches title, abstract, author keywords, and Keywords Plus.

Limits: none.

S.7.1. Primary Web of Science Core Collection search syntax developed for reproducibility

```
TS=(
  (
    "natriuretic peptide receptor C"
    OR "natriuretic peptide receptor-C"
    OR "natriuretic peptide receptor 3"
    OR "natriuretic peptide receptor-3"
    OR "natriuretic peptide receptor type C"
    OR "atrial natriuretic peptide receptor C"
    OR "atrial natriuretic peptide receptor-C"
    OR "atrial natriuretic peptide receptor 3"
    OR "atrial natriuretic peptide receptor type C"
    OR "atrionatriuretic peptide receptor C"
    OR "ANP receptor C"
    OR "ANP receptor-C"
    OR "ANF receptor C"
    OR "NP receptor C"
    OR "NP receptor-C"
    OR "natriuretic peptide clearance receptor"
    OR "atrial natriuretic peptide clearance receptor"
    OR "NPR-C"
    OR "NPR C"
    OR "NPR-3"
    OR NPRC
    OR NPR3
    OR ANPRC
    OR "ANPR-C"
    OR "ANP-C"
    OR (("clearance receptor" OR "clearance receptors") AND (natriuretic OR "atrial natriuretic" OR
"C-type natriuretic"))
    OR "C-type natriuretic peptide"
    OR "C type natriuretic peptide"
    OR NT-proCNP
    OR "NT proCNP"
    OR "amino-terminal pro-C-type natriuretic peptide"
    OR (CNP AND (natriuretic OR peptide* OR receptor*))
  )
  AND
  (
    "pulmonary hypertension"
    OR "pulmonary arterial hypertension"
    OR "group 3 pulmonary hypertension"
    OR "group III pulmonary hypertension"
    OR "pulmonary vascular disease"
    OR "pulmonary vascular remodeling"
    OR "pulmonary vascular remodelling"
    OR "pulmonary artery remodeling"
    OR "pulmonary artery remodelling"
    OR "pulmonary arterial remodeling"
    OR "pulmonary arterial remodelling"
    OR "pulmonary vascular resistance"
    OR "mean pulmonary arterial pressure"
    OR mPAP
    OR "right heart catheterization"
    OR "right heart catheterisation"
    OR "right ventricular"
    OR "right ventricle"
    OR "right ventricular dysfunction"
    OR "right ventricular hypertrophy"
    OR "right ventricular remodeling"
    OR "right ventricular remodelling"
    OR "cor pulmonale"
    OR "pulmonary artery smooth muscle"
    OR "pulmonary arterial smooth muscle"
    OR PASMC*
```

```
    OR "pulmonary endothelial"
    OR "pulmonary artery endothelial"
    OR "pulmonary arterial endothelial"
    OR PAEC
    OR monocrotaline
    OR SU5416
    OR Sugen
    OR "Sugen-hypoxia"
  )
  AND
  (
    "chronic lung disease"
    OR "chronic respiratory disease"
    OR COPD
    OR "chronic obstructive pulmonary disease"
    OR emphysema
    OR "interstitial lung disease"
    OR ILD
    OR "pulmonary fibrosis"
    OR "idiopathic pulmonary fibrosis"
    OR IPF
    OR "combined pulmonary fibrosis and emphysema"
    OR CPFE
    OR hypoxia
    OR hypoxic
    OR hypoxemia
    OR hypoxaemia
    OR "chronic hypoxia"
    OR "hypoxia-induced"
    OR "lung injury"
    OR bleomycin
    OR "cigarette smoke"
    OR "smoke exposure"
    OR "sleep apnea"
    OR "sleep apnoea"
    OR "sleep-disordered breathing"
    OR "sleep disordered breathing"
    OR hypoventilation
    OR "obesity hypoventilation"
    OR "bronchopulmonary dysplasia"
  )
)
```

S.7.2. Supplementary Web of Science Core Collection sensitivity search syntax developed for reproducibility

```
TS=(
  (
    "natriuretic peptide receptor C"
    OR "natriuretic peptide receptor-C"
    OR "natriuretic peptide receptor 3"
    OR "natriuretic peptide receptor-3"
    OR "natriuretic peptide receptor type C"
    OR "atrial natriuretic peptide receptor C"
    OR "atrial natriuretic peptide receptor-C"
    OR "atrial natriuretic peptide receptor 3"
    OR "atrial natriuretic peptide receptor type C"
    OR "atrionatriuretic peptide receptor C"
    OR "ANP receptor C"
    OR "ANP receptor-C"
    OR "ANF receptor C"
    OR "NP receptor C"
    OR "NP receptor-C"
    OR "natriuretic peptide clearance receptor"
    OR "atrial natriuretic peptide clearance receptor"
    OR "NPR-C"
    OR "NPR C"
    OR "NPR-3"
    OR NPRC
    OR NPR3
    OR ANPRC
    OR "ANPR-C"
    OR "ANP-C"
    OR (("clearance receptor" OR "clearance receptors") AND (natriuretic OR "atrial natriuretic" OR
"C-type natriuretic"))
```

```
      OR "C-type natriuretic peptide"
      OR "C type natriuretic peptide"
      OR NT-proCNP
      OR "NT proCNP"
      OR "amino-terminal pro-C-type natriuretic peptide"
      OR (CNP AND (natriuretic OR peptide* OR receptor*))
    )
    AND
    (
      "pulmonary hypertension"
      OR "pulmonary arterial hypertension"
      OR "pulmonary vascular disease"
      OR "pulmonary vascular remodeling"
      OR "pulmonary vascular remodelling"
      OR "pulmonary artery remodeling"
      OR "pulmonary artery remodelling"
      OR "pulmonary arterial remodeling"
      OR "pulmonary arterial remodelling"
      OR "pulmonary vascular resistance"
      OR "mean pulmonary arterial pressure"
      OR mPAP
      OR "right heart catheterization"
      OR "right heart catheterisation"
      OR "right ventricular"
      OR "right ventricle"
      OR "right ventricular dysfunction"
      OR "right ventricular hypertrophy"
      OR "right ventricular remodeling"
      OR "right ventricular remodelling"
      OR "cor pulmonale"
      OR "pulmonary artery smooth muscle"
      OR "pulmonary arterial smooth muscle"
      OR PASMC*
      OR "pulmonary endothelial"
      OR "pulmonary artery endothelial"
      OR "pulmonary arterial endothelial"
      OR PAEC
      OR monocrotaline
      OR SU5416
      OR Sugen
      OR "Sugen-hypoxia"
    )
  )
```

S.8. PRISMA-S reporting wording for manuscript methods

Manuscript reporting wording: Electronic searches were conducted in PubMed/MEDLINE and APA PsycINFO via APA PsycNet from database inception to 18 May 2026. A Web of Science Core Collection strategy was developed for reproducibility and is reported in Appendix A, but this database search was not executed and was not included in the final PRISMA counts. The search strategy combined controlled vocabulary and free-text terms for natriuretic peptide receptor-C/NPR3 biology, pulmonary hypertension or pulmonary vascular/right ventricular disease, and chronic lung disease or hypoxia-related lung disease. Database-specific field tags and syntax were used, including MeSH and [tiab] terms in PubMed/MEDLINE and Any Field searching in APA PsycINFO via APA PsycNet. No restrictions were applied by date, language, publication type, age group, or species. Reviews and other non-original records were not excluded at the search stage; they were retained for citation tracking, while final eligibility was restricted to original empirical studies according to the review protocol. Full search strategies are provided in Supplementary File S2. Records retrieved from PubMed/MEDLINE and APA PsycINFO were exported, deduplicated, and screened according to the PRISMA-ScR study selection process.

S.9. Source notes for search syntax

1. Rethlefsen ML, Kirtley S, Waffenschmidt S, Ayala AP, Moher D, Page MJ, Koffel JB, PRISMA-S Group. PRISMA-S: an extension to the PRISMA Statement for Reporting Literature Searches in Systematic Reviews. Systematic Reviews. 2021;10:39. doi:10.1186/s13643-020-01542-z. https://doi.org/10.1186/s13643-020-01542-z
2. National Library of Medicine. PubMed Help: search field tags and PubMed syntax. https://pubmed.ncbi.nlm.nih.gov/help/
3. American Psychological Association. APA PsycNet Quick Reference Guide. https://www.apa.org/pubs/databases/training/psycnet.pdf

4. Clarivate. Web of Science Core Collection Advanced Search Field Tags. https://webofscience.zendesk.com/hc/en-us/articles/26916347018257-Web-of-Science-Core-Collection-Advanced-Search-Field-Tags
